# A Redundancy Metric Set within Possibility Theory for Multi-Sensor Systems †

**DOI:** 10.3390/s21072508

**Published:** 2021-04-03

**Authors:** Christoph-Alexander Holst, Volker Lohweg

**Affiliations:** inIT–Institute Industrial IT, Technische Hochschule Ostwestfalen-Lippe, Campusallee 6, 32657 Lemgo, Germany; volker.lohweg@th-owl.de

**Keywords:** redundancy analysis, possibility theory, multi-sensor systems, information fusion

## Abstract

In intelligent technical multi-sensor systems, information is often at least partly redundant—either by design or inherently due to the dynamic processes of the observed system. If sensors are known to be redundant, (i) information processing can be engineered to be more robust against sensor failures, (ii) failures themselves can be detected more easily, and (iii) computational costs can be reduced. This contribution proposes a metric which quantifies the degree of redundancy between sensors. It is set within the possibility theory. Information coming from sensors in technical and cyber–physical systems are often imprecise, incomplete, biased, or affected by noise. Relations between information of sensors are often only spurious. In short, sensors are not fully reliable. The proposed metric adopts the ability of possibility theory to model incompleteness and imprecision exceptionally well. The focus is on avoiding the detection of spurious redundancy. This article defines redundancy in the context of possibilistic information, specifies requirements towards a redundancy metric, details the information processing, and evaluates the metric qualitatively on information coming from three technical datasets.

## 1. Introduction

Multi-sensor systems exhibit redundancy inherently. This is especially true for intelligent technical or cyber–physical systems (CPS)—such as industrial production systems, power plants, transportation vehicles, or even technical mobile devices [1,2]. Sensors are either intentionally designed to be redundant or redundancy inherently emerges due to interrelated dynamic processes. For example, temperature, electric current, and frequency characteristics of an electric motor may all be affected by damages to the motor’s bearing and, thus, may provide redundant information in the context of the motor’s condition. Redundancy allows a multi-sensor system to be more robust against sensor defects, environmental influences, or outlier measurements. It acts as a fail-safe to ensure that a system remains continuously and fully operational. Redundancy comes with a cost—both computationally and regarding the complexity of models. Knowing which sensors are redundant or at least partly redundant allows to explicitly exploit the redundancy to make a system more robust or to actively avoid computational costs. Determining which sensors are redundant as well as quantifying the degree of redundancy is in large multi-sensor systems no trivial task. This task of redundancy analysis is addressed both in information fusion and machine learning methods.

Information fusion aims at reducing uncertainties by aggregating information from multiple sensors or sources [3,4]. In addition to reducing uncertainty, redundant information allows a fusion system both to increase its robustness and to identify unreliable, drifting, or malfunctioning sensors [5,6,7,8]. Designing an information fusion system involves the decision of which sensors are to be fused at which stage in the information processing. Sensors are usually grouped manually by their information quality, spatial proximity, or semantic proximity such as in [9,10,11]. More generally, sensors are grouped by their expected redundant behaviour. In modern systems consisting of large amounts of sensors and other information sources, a manual approach is not feasible. Identifying redundant sensors automatically from training data benefits information fusion system design. In machine learning, redundancy is either taken advantage of implicitly, for instance random forests, or identified (and removed) explicitly, such as in the field of feature selection. In feature selection, redundant information is conceived as unnecessary burden for the training of the machine learning model. Redundant features increase computational costs and difficulty of the learning task without providing new information [12]. Thus, quantifying the redundancy between features is beneficial in this field also.

**Figure 1:**
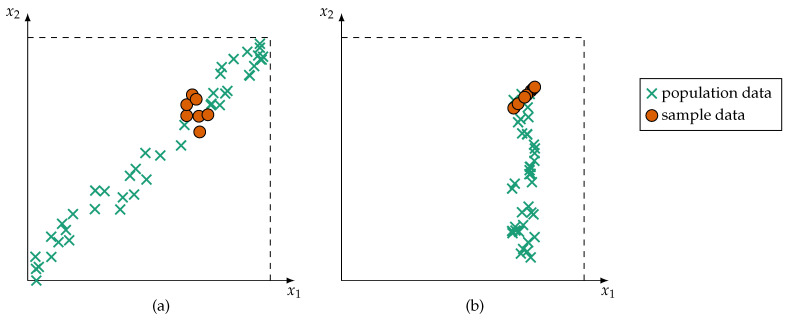
Examples of variables $x_{1},x_{2}\in R$ showing (**a**) similar behaviour which is not apparent in the sample data and showing (**b**) non-similar behaviour although sample data indicate otherwise (which is an example of spurious correlation). These kinds of biased or skewed sample data commonly occur, for example, in production systems. Production systems execute tasks repetitively in a normal (as in functioning properly) condition. In this case, data are not sampled randomly and do not match the population distribution.

Intelligent technical or cyber–physical systems make it particularly challenging to identify redundancies. In these systems, sensors may be unreliable and information is often affected by aleatoric and epistemic uncertainties. Aleatoric uncertainties are characterized by non-deterministic, random processes which can be modelled statistically, such as noise. Epistemic uncertainties stem from a lack of information, imprecision, or bias. Such incomplete information manifests itself at two levels:

- At the level of single sensor measurements, lack of information, e.g., about the sensor’s detailed characteristics, tolerances, or physical limits, results in imprecise readings. Thus, a sensor is only able to give an approximate measurement. As a result of this, information is often provided in intervals, fuzzy intervals, or uncertainty distributions (either probabilistic or possibilistic) [13].
- Furthermore, during training, the monitored process may only by observable in specific states. For example, a production machine may create a lot of training data, but these data often originate from the same machine state, that is, data about states of failure are rare. This leads to ambiguous and fuzzy classes [14] as well as premature detection of interrelations (such as redundancy) between sensors. The risk of detecting spurious correlations [15] is greatly amplified in intelligent technical or cyber–physical systems. Two examples of premature detection of variable interrelation are shown in Figure 1.

This contribution proposes a metric for quantifying redundancy intended for the application in technical or cyber–physical multi-sensor systems. It is a continuation and extended work of a conference contribution published in [16]. To cope with incomplete information, the proposed redundancy metric is embedded in the framework of possibility theory. Possibility theory is specifically conceived to represent and handle imprecise information. In this article, it is presented and discussed how possibilistic measures, such as similarity, specificity, or consistency, fit in and contribute to a possibilistic redundancy metric. A focus is on avoiding premature detection of spurious relations. Only if sufficient evidence is available that the information does not originate from the same repetitive process state, does the metric indicate redundancy so that further data processing algorithms are not impeded negatively. Otherwise, machine learners would be deprived of crucial information and information fusion systems would detect reliable sensors as unreliable.

In the remainder of this contribution, single pieces of information are referred to as *information items* which are provided by an *information source* (nomenclature after Dubois et al. [4]).

**Definition** **1** (**Information Item**)**.** *Consider an unknown entity v and a non-empty set of possible alternatives $X_{A}=\left\{x_{1},\ldots ,x_{n}\right\}$ with $n\in N_{>0}$. An information item models information in the form of plausibilities or probabilities about v regarding $X_{A}$. An information item can, e.g., be a set, an interval, a probability distribution, or a possibility distribution. Consequently, an item may be expressed with certainty (v=x or, assuming $A\subset X_{A}$, v∈A), may be affected by uncertainty (v is probably x or v is possibly x), or may be expressed imprecisely ($x_{1}<v<x_{2}$).*

**Definition** **2** (**Information Source**)**.** *An information source S provides information items. It is an ordered concatenation of information items $S=\{I_{1},I_{2},\ldots ,I_{m}\}$ with $m\in N_{>0}$. Each $I_{j}$ represents an information item at instance j∈{1,…,m}. In case of multiple information sources, indexing is applied as follows: Let $S_{i}$ with $i\in N_{>0}$ be an information source, then its information items are indexed with $I_{i,j}$. An information source may be, for example, a technical sensor, a variable, a feature, or a human expert.*

The following Section 2 reviews definitions of redundancy in the state-of-the-art and gives an overview of how redundancy is quantified in related work. Section 3 recaptures the fundamentals of possibility theory and discusses both differences and advantages with regard to probability theory. The proposed possibilistic redundancy metric is then detailed in Section 4. In Section 5, the redundancy metric is implemented on several technical datasets and qualitatively evaluated. A conclusion and an outlook are given in Section 6.

## 2. Redundancy in Related Work

In order to be able to quantify redundancy between sources, a precise definition of redundancy is required first. The use of the term redundancy across scientific works and literature of different fields carries often slight variations in meaning (partly due to the vague linguistic use of the term redundancy). Although the focus of related work is often on actively reducing redundancy in sets of features, variables, data, or sensors, redundancy itself is often only referred to implicitly, e. g., in [12,17,18]. Only rarely is redundancy defined explicitly. One of the earliest and fundamentally important explicit definitions of redundancy is given within the information theory [19] in which redundancy is defined as the difference between the maximum possible information content in a transmission and its actual transmitted content [20]. Redundancy occurs, here, due to transmitted symbols which carry information already present in the message.

In further scientific works and fields, two slightly different interpretations of redundancy can be distinguished. In their paper regarding fuzzy rule systems, Lughofer and Hüllermeier [21] touch the issue of two interpretations and state that redundancy can either be reflected by “inclusion” or “similarity”. Inclusion means that a piece of information is deemed as redundant if, and only if, it does not contribute or add new information to an already existing state of knowledge—it is included in already known information. The notion of similarity refers to information items or sources which are exchangeable with each other.

Works focusing on knowledge bases, fuzzy rule bases, or association rule mining often define redundancy with respect to inclusion. Dubois et al. [22] define redundancy in the context of fuzzy knowledge bases. According to their work, an information item, represented by a fuzzy set or possibility distribution, is regarded as redundant iff an already known information item is not changed by combining both items. Similarly, Dvořák et al. [23] present an example of a redundant fuzzy rule stating that a rule is redundant if their antecedent is covered (included) by another rule (and both rules have the same consequences). Bastide et al. [24] and Díaz Vera et al. [25] specify within association rule mining that a rule is redundant “if it conveys the same information—or less general information—than the information conveyed by another rule”. Zhang et al. [26] define in the context of document analysis that a document is redundant if all relevant information is already covered in previous documents. From these considerations, it can be gathered that the first type of redundancy is directional dependent, i.e., if an information item is redundant with regard to a second item, then it does not follow that the second one is redundant with regard to the first item. In the following, this form of redundancy is referred to as Redundancy Type I.

Similarity as a measure of redundancy can often be found in works regarding information fusion or feature selection. In information fusion as well as sensor fusion, redundant information results from information sources monitoring the same objects, concepts, or features in an environment [1,17]. By perceiving or measuring the same properties independently, sources provide similar pieces of information. In [10,11,27], condition monitoring fusion systems applied to technical machines are manually orchestrated and designed so that sensors are fused which observe the same parts of a machine. In this way, the emerging redundancy is exploited to handle conflicts between sensor readings. Interpreting redundancy as similarity between information sources is also dominantly found in the field of feature selection. For example, Auffarth et al. [28] write that “redundancy measures how similar features are”. Chakraborty et al. [29] and Pfannschmidt et al. [30,31] argue that features or variables include redundancy if not all relevant features are required for a target application, that is, there exists no unique minimum feature set to solve a given task. This kind of redundancy, based on similarity of information, is in this work hereafter referred to as Redundancy Type II.

There have been multiple approaches proposed to determine or measure the redundancy of information sources based on their similarity—extensively within the probability theory. Multiple works state that redundant information sources are highly correlated [32,33]. Yu and Liu [34] report furthermore that it is widely accepted that information sources are regarded as redundant if they are perfectly linearly correlated. Thus, Hall [33] makes use of the Pearson’s correlation coefficient to measure the redundancy between information sources. The term correlation-based feature selection goes back to the doctoral dissertation of Hall. Several papers build upon the correlation-based feature selection to improve implementations and fasten the search for redundancies in sets of sources such as [35,36]. More recent applications of correlation as a redundancy measure can be found in [8,37,38,39]. Goswami et al. [37] cluster features based on their redundancy determined using the Pearson’s correlation coefficient. In [38,39], redundant features are eliminated based on correlation coefficients for applications in biology, whereas Berk et al. [8] determine reliability and redundancy of sensors in an automated driving scenario. However, there has been some debate in the feature selection community about the appropriateness of using correlation-based metrics. Guyon et al. [12] argue that correlation does not imply redundancy. They give simple examples where two features are highly correlated but both are clearly required to solve a classification task.

Another popular method to measure Redundancy Type II probabilistically is mutual information (MI) based on the information theory. Battiti et al. [40] apply MI both as a measure for redundancy as well as relevance of features. Of particular note is the minimum redundancy–maximum relevance selection algorithm proposed by Ding and Peng [18,41] which incorporates mutual information for quantifying redundancy. MI is more recently applied as a redundancy measure in [42] and extended to work with multi-label feature selection [43,44] or non-linear data [45]. Mutual information is based on the entropy of a random variable and requires knowledge about the underlying probability distribution of data. This knowledge is in technical systems often hard to obtain. Mutual Information, unlike Pearson’s correlation coefficient, does not assume a linear correlation between features, i.e., it is able to detect redundancy if data are non-linearly correlated. However, both the correlation coefficient and MI assume that information is available as precise singleton values. They are not readily applicable to information which is imprecise or vague such as information modeled with an uncertainty distribution—probabilistic or possibilistic.

Works which address redundancy between information sources outside the probability framework are comparatively rare. Methods that come closest to quantifying redundancy, such as [5,46,47,48], identify non-redundancy in a group of information sources. These methods assume that sources are at least partly redundant and, based on this assumption, aim to detect unreliable sources which are characterized by non-redundant behaviour such as inconsistencies. Both Ricquebourg et al. [46,47] and Ehlenbröker et al. [5] monitor streaming data to identify unreliable sources either by quantifying (i) their degree of conflict based on the Dempster–Shafer theory or (ii) their degree of inconsistency based on the possibility theory. Since both methods only identify non-redundant behaviour, they cannot readily be considered as redundancy metrics.

In the remainder of this paper the focus is on Redundancy Type II (based on similarity of information) since multi-sensor systems for machine analysis exploit this kind of redundancy—as described or applied in [1,10,11,17,27]. Nonetheless, Redundancy Type I (information is evaluated against already known information) is discussed wherever necessary or appropriate.

## 3. Possibility Theory

The possibility theory (PosT) was introduced by Zadeh [49] in 1978 motivated by the observation that probability theory (ProbT) handles epistemic uncertainty only insufficiently. Zadeh defines PosT as an extension of fuzzy sets in the sense that possibility distributions allow uncertainties (meaning as a statement of confidence or lack thereof) within fuzzy information of natural language [50]. Therefore, fuzzy set theory has the same relation to PosT as the measurement theory to ProbT, that is, crisp sets and random variables are the natural variables of ProbT while fuzzy sets and fuzzy numbers are the natural variables of PosT [51]. Since its first introduction, the possibility theory has been extensively advanced by Dubois and Prade (e.g., in [4,13,52,53,54,55]) and Yager (e.g., in [56,57,58,59,60]), among others. In the following, we assume a numerical, real-valued representation of possibility scales because we focus on measurements in multi-sensor systems (cf. [4] for an overview of qualitative and numerical possibility scales).

### 3.1. Basics of Possibility Theory

Let *X* be a set of mutually exclusive and exhaustive alternative events, i.e., the entirety of possible events, then *X* is referred to as the universe of discourse or frame of discernment [61]. Let v∈X be an existing but unknown or imprecisely known element—the true value of *v* is unknown. Then a possibility distribution is a mapping

(1) $$\pi_{v}:X\to \left[0,1\right].$$

If $\pi_{v}\left(x\right)>\pi_{v}\left(x^{\prime }\right)$, then v=x is more plausible than $v=x^{\prime }$. A possibility of $\pi_{v}\left(x\right)=0$ means that it is impossible that v=x. The case of $\pi_{v}\left(x\right)=1$ is interpreted that there is no evidence preventing v=x, i.e., *x* is a completely plausible value for v=x. Possibility distributions allow to model two extreme cases of knowledge. Total ignorance exists if nothing is known about *v*—all alternatives are fully possible, i.e., $\forall x\in X:\pi_{v}(x)=1$. The other extreme situation in which only a single unique alternative $x_{0}$ is completely possible and all other alternatives are impossible, i.e., $\pi (x_{0})=1$ and $\forall x\in \left\{X\backslash x_{0}\right\}:\pi \left(x\right)=0$ is referred to as complete knowledge. A possibility distribution is said to be normal if, for a subset A⊆X, $\exists x\in A:\pi_{v}\left(x\right)=1$.

There exists a special relationship between possibility distributions and membership functions of fuzzy sets (μ:X→[0,1]) [49]. A membership function can readily serve as a possibility distribution although the interpretation of both is different [62]. Fuzzy membership functions convey a degree of truth, whereas possibility distributions convey a degree of certainty (confidence) [50]. This is helpful in practical implementations because mathematical operations defined in the context of fuzzy sets—such as similarity measures or t-norms—can often be applied to possibility distributions.

**Figure 2:**
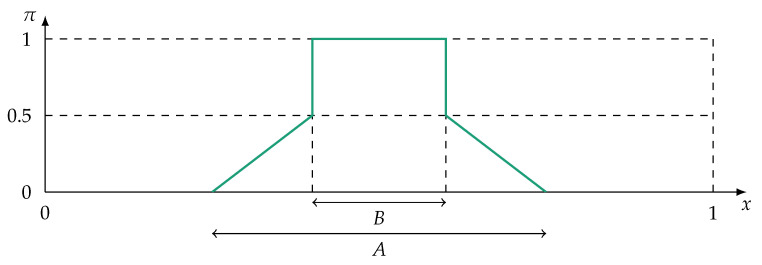
A possibility distribution $\pi_{v}$. For any element x∈B, v=x is fully plausible; for $x\in (A\cap B^{c})$, v=x is only partially plausible; and for $x\in A^{c}$, v=x is impossible. The accompanying possibility and necessity measures for A,B are: Π(A)=1, N(A)=1 and Π(B)=1, N(B)=0.5.

An example of a possibility distribution is given in Figure 2. Note that outside of this section, the shortened notation $\pi \left(x\right)=\pi_{v}\left(x\right)$ is used.

Based on possibility distributions, the possibility and necessity of a crisp set can be determined by two dual possibilistic set functions. Given two crisp sets A,B⊆X and the complement set $A^{c}$, the possibility measure and necessity measure are defined by

$$\Pi \left(A\right)=\underset{x\in A}{\sup}\pi_{v}\left(x\right),\;N\left(A\right)=1-\Pi \left(A^{c}\right)=\underset{x\notin A}{\inf}\left(1-\pi_{v}\left(x\right)\right),$$

respectively [52]. Possibility theory is then defined axiomatically as an independent theory by

Π(⌀)=0,

(2) Π(X)=1,

and the maxitivity axiom

Π(A∪B)=max(Π(A),Π(B))

in contrast to the additivity axiom of probability theory.

### 3.2. Possibility Theory in Comparison to Probability Theory

The main difference between possibility theory and probability theory is that ProbT models random phenomena quantitatively whereas PosT models incomplete information qualitatively. Possibility theory is specifically designed to handle epistemic uncertainties such as missing, imprecise, or sparse information [63]. On the other hand, the presence of only incomplete information is precisely the situation in which the probability of an event is ill-known [62]. This argument motivates the proposition of this paper: to embed a redundancy metric which functions in poorly informed scenarios. Specifically, our contribution draws upon advantages of PosT over ProbT such as:

- The application of PosT does not require statistical data to be available. Consequently, it is easier and takes less effort to construct sound possibility distributions than probability distributions (cf. [54] for methods to construct possibility distributions).
- In contrast to ProbT, both imprecision and confidence can be modelled distinctly within a possibility distribution. Imprecision is modeled by allowing multiple alternatives to be possible, e.g., it may be known that v∈A, but not which value *v* takes within *A* precisely. Confidence is expressed by the degree of possibility assigned to a value *x*, i.e., if $0<\pi_{v}\left(x\right)<1$, it is uncertain if v=x is fully possible. It follows directly that confidence is also represented in the duality measure of Π and *N* as can be seen in the three extreme epistemic situations [50]: (i) if v∈A is certain, $\Pi_{v}\left(A\right)=1$ and $N_{v}\left(A\right)=1$, (ii) if v∉A is certain, $\Pi_{v}\left(A\right)=0$ and $N_{v}\left(A\right)=0$, and (iii) in case of ignorance, $\Pi_{v}\left(A\right)=1$ and $N_{v}\left(A\right)=0$.

Nonetheless, as pointed out by Dubois et al. in [63], PosT is a complementary alternative to ProbT but not a general substitute. If sound statistics are available—which is in technical systems often not the case—then probabilistic approaches are to be preferred. Even if probabilistic uncertainty distributions are available, possibilistic methods can still be applied with the help of probability-possibility transforms [53,64,65,66,67]. Since possibilistic representations are inherently imprecise, they convey less information than a probability distribution. It follows that in a transform information is lost. In applying probability-possibility transforms it has to be kept in mind that, because of this loss of information, there is no inverse transformation.

### 3.3. Fusion within Possibility Theory

Consider several information sources $\left\{S_{1},\ldots ,S_{n}\right\}$ which all provide an information item in the form of $\pi_{i}$, i∈{1,…,n} about the same unknown element *v* in the same frame of discernment *X*. Information fusion is then carried out by a function $\mathrm{fu}:{\left[0,1\right]}^{n}\to \left[0,1\right]$. The aim of information fusion in general is to produce information of higher quality [2]. In the context of possibility theory, fusion is driven by the minimum specificity principle, i.e., any hypothesis which is not explicitly known to be impossible must not be rejected [50].

In PosT, there are several approaches towards the fusion of possibility distributions [61,63]. Deciding which method is the most appropriate depends on the consistency of information in $\left\{\pi_{i}\right\}$, the reliability of the available information, and the knowledge which specific $S_{i}$ is not reliable. Consistency within a group of possibility distributions is formally defined [13] as

(3) $$h(\pi_{1}\left(x\right),\ldots ,\pi_{n}\left(x\right))=\max_{x\in X}(\min_{i\in \{1,\ldots ,n\}}\left(\pi_{i}(x)\right)).$$

The different approaches, then, are:

- Conjunctive fusion modes implement the principle of minimal specificity most strongly. By applying a triangular norm (t-norm),
  $$\pi^{\left(\mathrm{fu}\right)}=\mathrm{fu}\left(\pi_{1}\left(x\right),\ldots ,\pi_{n}\left(x\right)\right)=t\left(\pi_{1}\left(x\right),\ldots ,\pi_{n}\left(x\right)\right),$$
  conjunctive fusion reduces the information to alternatives all sources can agree on. An overview of t-norms, and their counterpart s-norms (also referred to as t-conorms), can be found in [68]. If at least one source is inconsistent with the remaining sources, i.e., the sources cannot agree on a fully plausible alternative, then the fused possibility distribution is subnormal ($\max(\pi^{\left(\mathrm{fu}\right)})<1$) or even empty. This violates the axiom (2) of PosT that at least one alternative in *X* must be fully plausible. A renormalisation
  (4) $$\pi^{\left(\mathrm{fu}\right)}=\frac{t\left(\pi_{1}\left(x\right),\ldots ,\pi_{n}\left(x\right)\right)}{h\left(\pi_{1}\left(x\right),\ldots ,\pi_{n}\left(x\right)\right)}$$
  prevents subnormal fusion results, but is numerically unstable if at least one source is fully inconsistent, i.e., $h(\pi_{1}\left(x\right),\ldots ,\pi_{n}\left(x\right))=0$.
- In case of fully inconsistent possibility distributions at least one information source must be unreliable. Assuming it is not known which source is unreliable, disjunctive fusion modes apply s-norms so that as much information is kept as possible:
  (5) $$\pi^{\left(\mathrm{fu}\right)}=s\left(\pi_{1}\left(x\right),\ldots ,\pi_{n}\left(x\right)\right).$$
  Disjunctive fusion is generally not desirable because the fusion does not result in more specific information.
- Adaptive fusion modes combine conjunctive and disjunctive fusion methods. These modes switch from conjunctive to disjunctive aggregation depending on which of the alternatives the sources are inconsistent for. An adaptive fusion mode, proposed in [69], is
  (6) $$\pi^{\left(\mathrm{fu}\right)}=\max(\frac{t(\pi_{1}\left(x\right),\ldots ,\pi_{n}\left(x\right))}{h(\pi_{1}\left(x\right),\ldots ,\pi_{n}\left(x\right))},\min(1-h(\pi_{1}\left(x\right),\ldots ,\pi_{n}\left(x\right))),s(\pi_{1}\left(x\right),\ldots ,\pi_{n}\left(x\right))).$$
  Thus, fusion results in a global level of conflict (1−h(·)) for all alternatives the sources cannot agree on. Otherwise the adaptive fusion reinforces by conjunction.
- A majority-guided fusion searches for the alternatives which are supported by most sources. This is similar to a voting style consensus. Majority-guided fusion requires the identification of a majority subset—usually the subset with highest consistency and maximum number of sources. The possibility distributions of this subset are fused conjunctively. Information outside of the majority subset is discarded which violates the fairness principle postulated in [4]. Applications of majority-guided fusion can be found in previous works of the authors of this contribution [6,7].

**Figure 3:**
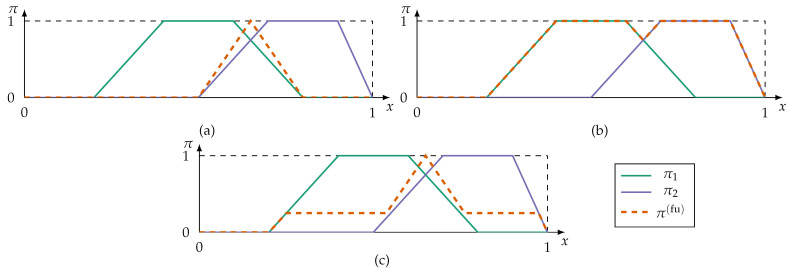
Different fusion approaches in possibility theory. Part (**a**) shows conjunctive fusion (4) using the minimum operator as t-norm, (**b**) illustrates disjunctive fusion (5) using the maximum operator as s-norm, and (**c**) shows the adaptive fusion rule (6) presented in [69] (also relying on minimum and maximum operators).

Conjunctive, disjunctive, and adaptive fusion are exemplary shown in Figure 3.

## 4. Quantifying Redundancy within the Possibility Theory

Redundancy metrics, such as Pearson’s correlation coefficient or mutual information, are not able to handle epistemic uncertainty or incomplete information intrinsically. In this section, possibilistic redundancy metrics for information sources as well as information items are proposed which fill this gap. These metrics are designed (i) to be able to process imprecise data affected with uncertainty distributions and (ii) to not detect spurious redundancy. They are intended to be favourable in applications in which information is systemically scarce, incomplete, or biased, such as in intelligent technical multi-sensor systems.

Since the redundancy of information sources is based on the redundancy of their information items, the latter are formalized first in Section 4.1. Following this, it is presented in Section 4.2 how the single redundancy assessments of items are combined to an overall redundancy metric. The two types of incomplete information, as introduced in Section 1, are addressed in this two step procedure. Lack of information at the sensor measurement level (uncertainty distributions) is covered on information item level, whereas incomplete information caused by biased or skewed data (see Figure 1) is dealt with on information source level. In addition to incomplete information, the effects of unreliable information on the redundancy metric are discussed. Especially in large multi-sensor systems, it is likely that unreliable information sources are present. It is, therefore, advantageous for a redundancy metric if it is robust against such unreliable or sporadically unreliable information sources (similar as fusion methods consider unreliable sources as described in Section 3.3).

### 4.1. Redundant Information Items

Information items can either be type I or type II redundant (see Section 2). Redundancy Type I and Redundancy Type II are defined and discussed separately. In the following, information items are provided as possibility distribution, i.e., I=π.

**Definition** **3** (**Redundancy Type I**)**.** *An information item is type I redundant if the carried information is already included in previously known information. Given an information item I and an unordered set of information items $\left\{I_{1},\ldots ,I_{n}\right\}$ with $n\in N_{>0}$, a possibilistic redundancy metric $r^{\left(I\right)}\left(I,\left\{I_{1},\ldots ,I_{n}\right\}\right)$ quantifies the degree of redundancy of I towards $\left\{I_{1},\ldots ,I_{n}\right\}$. A metric for Redundancy Type I satisfies the following properties:*

- **Boundaries:** Information items can be minimally and maximally redundant. Therefore, $r^{\left(I\right)}$ is minimally and maximally bounded: $r^{\left(I\right)}\in \left[0,1\right]$.
- **Inclusion (Upper Bound):** An information item $I_{1}$ is fully redundant in relation to $I_{2}$ if it encloses (includes) $I_{2}$.
- **Lower Bound:** An information item is non-redundant if it adds new information. Additionally, an item $I_{1}$ is fully non-redundant in relation to $I_{2}$ if $I_{1}$ and $I_{2}$ disagree completely on the state of affairs, i.e., in terms of possibility theory $h\left(\pi_{1},\pi_{2}\right)=0$.
- **Identity:** Two identical information items are fully redundant, i.e., $r^{\left(I\right)}(I,I)=1$.

*Redundancy Type I is not bidirectional or symmetric, i.e., if $r^{\left(I\right)}\left(I_{1},I_{2}\right)>0⇏r^{\left(I\right)}\left(I_{2},I_{1}\right)>0$.*

**Definition** **4** (**Redundancy Type II**)**.** *Information items are type II redundant if they convey similar information with regard to a given task. This given task can be solved relying on any one of the information items. Let I be a set of unordered information items and P(I) all possible combinations of information items, then Redundancy Type II is a function $r^{\left(\mathrm{II}\right)}:P(I)\to \left[0,1\right]$. Similarly to $r^{\left(I\right)}$, $r^{\left(\mathrm{II}\right)}$ is required to satisfy the properties of boundaries and identity as defined in Definition 3. Additionally, it has the following properties:*

- **Symmetry:** A redundancy metric $r^{\left(\mathrm{II}\right)}$ is symmetric in all its arguments, i.e., $r^{\left(\mathrm{II}\right)}(I_{1},I_{2},\ldots ,I_{n})=r^{\left(\mathrm{II}\right)}(I_{p(1)},I_{p(2)},\ldots ,I_{p(n)})$ for any permutation p on $N_{>0}$.
- **Non-Agreement (Lower Bound):** Information items are fully non-redundant if they disagree completely on the state of affairs, i.e., they do not agree on at least one alternative in the frame of discernment to be possible, i.e., $h(\pi_{1},\pi_{2})=0$.

#### 4.1.1. Redundancy Type I

An information item represented by a possibility distribution $\pi_{1}$ is completely type I redundant iff it includes the previously known information $\pi_{2}$ [22]. This notion stems originally from the fuzzy set theory. In this context, a fuzzy set *A* includes another set *B* iff B⊆A. Relying on the mathematical closeness between fuzzy memberships and possibility degrees (μ=π), complete redundancy is then determined as follows:

(7) $$\begin{array}{c} r^{\left(I\right)}\pi_{1},\pi_{2}=\left\{\begin{array}{cc} 1 & \mathrm{if}\;\mathrm{and}\;\mathrm{only}\;\mathrm{if}\;\forall x\in X:\pi_{2}\left(x\right)\leq \pi_{1}\left(x\right),\\ 0 & \mathrm{otherwise}. \end{array}\right. \end{array}$$

This formalization of a Redundancy Type I measure determines whether an information item is completely redundant or not at all ($r^{\left(I\right)}=1$ or $r^{\left(I\right)}=0$). As soon as a possibility distribution does not completely include the already known distribution, it is regarded as completely non-redundant. For practical purposes in information fusion and multi-sensor systems, it is helpful to determine grades of redundancy. In the following a metric of type I is proposed which uses the real-valued, continuous space [0,1].

This metric is based on the notion that information is altered (preferably: gained) by considering and fusing an additional possibility distribution. Due to the additional consideration of $\pi_{1}$, the fused possibility distribution obtained by $\mathrm{fu}(\pi_{1},\pi_{2})$ has a different uncertainty than $\pi_{2}$. It is more or less specific. The specificity of a possibility distribution is a measure of its information content. The more specific π, the more information is contained in π [61]. Specificity has been addressed by Zadeh [49], Dubois et al. [53], and Mauris et al. [66] as a relative quantity between two information items ($\pi_{1}$ is more specific than $\pi_{2}$ if $\forall x\in X:\pi_{1}\left(x\right)<\pi_{2}\left(x\right)$). Measures which determine specificity quantitatively have been proposed by Yager [57,58,60] and Higashi and Klir [70,71].

According to Yager, a specificity measure spec(π)∈[0,1] has to satisfy four conditions:

1. spec(π)=0 in case of total ignorance, i.e., ∀x∈X:π(x)=1.
2. spec(π)=1 iff in case of complete knowledge, i.e., only one unique event is totally possible and all other events are impossible.
3. A specificity measure de- and increases with the maximum value of π(x), i.e., let $\pi_{k}$ be the *k*th largest possibility degree in π(x), then $\frac{\;d\mathrm{spec}(\pi )}{\;d\pi_{1}}>0$.
4. $\forall k>2:\frac{\;d\mathrm{spec}(\pi )}{\;d\pi_{k}}\leq 0$, i.e., the specificity decreases as the possibilities of other values approach the maximum value of π(x).

An uncertainty measure u(π)∈[0,1] is then an order reversing one-to-one mapping of spec with u(π)=1 if spec(π)=0. In [70] the reverse mapping is obtained by u(π)=1−spec(π). These measures of possibilistic uncertainty and possibilistic specificity are the counterpart of Shannon’s probabilistic entropy [50,70].

Based on [71], the gain of information $g:{\left[0,1\right]}^{2}\to \left[0,1\right]$ when a possibility distribution $\pi_{2}$ is replaced by $\pi_{1}$ is

(8) $$g\left(\pi_{1},\pi_{2}\right)=u\left(\pi_{2}\right)-u\left(\pi_{1}\right).$$

The information gain quantifies the loss of uncertainty or gain in specificity. If $g(\pi_{1},\pi_{2})<0$, then by replacing $\pi_{2}$ with $\pi_{1}$ uncertainty is increased.

Measures of possibilistic uncertainty interpret possibility distributions as fuzzy sets and make use of fuzzy set α-cuts. Let A⊆X and the set $A_{\alpha }$ be the crisp subset of *A* which contains all elements *x* for which π(x)≥α with α∈[0,1]. In this way, an α-cut operator reduces a fuzzy set to a crisp set. An uncertainty measure for discrete frame of discernments based on [71] is

$$u(\pi )=\frac{1}{\log_{2}(\left|X\right|)}\cdot \int_{0}^{\alpha_{\max}}\log_{2}(|A_{\alpha }|)\;d\alpha$$

in which |A| denotes the cardinality of set *A* and $\alpha_{\max}=\max_{x\in A}\left(\pi \left(x\right)\right)$. A measure of specificity for real-valued, continuous frame of discernments is given in [57,58,60]:

(9) $$\mathrm{spec}\left(\pi \right)=\alpha_{\max}-\frac{1}{\left(x_{b}-x_{a}\right)}\cdot \int_{0}^{\alpha_{\max}}\left(\max_{x\in A_{\alpha }}x-\min_{x\in A_{\alpha }}x\right)\;d\alpha ,$$

with $x_{a}$ and $x_{b}$ being the borders of *X* ($X=[x_{a},x_{b}]$). For (9), it is proven in [57,58,60] that the measure satisfies the four requirements for specificity measures. The integral in (10) is equivalent to the area under *A* [56]. Therefore, (9) is equal to

(10) $$\begin{array}{cc} \mathrm{spec}(\pi ) & =\alpha_{\max}-\frac{1}{\left(x_{b}-x_{a}\right)}\cdot \int_{x_{a}}^{x_{b}}\pi (x)\;dx\\ \; & =\max_{x\in X}\pi (x)-\frac{1}{\left(x_{b}-x_{a}\right)}\cdot \int_{x_{a}}^{x_{b}}\pi (x)\;dx. \end{array}$$

Relying on the specificity measure in (10), the information gain defined in (8) is the basis of the proposed Redundancy Type I measure so that

(11) $$r^{\left(I\right)}(\pi_{1},\pi_{2})=\left(1-|g(\mathrm{fu}(\pi_{1},\pi_{2}),\pi_{2})|\right)\cdot h(\pi_{1},\pi_{2}),$$

i.e., the gain of information by fusing $\pi_{1}$ and $\pi_{2}$ is the basis of $r^{\left(I\right)}$. The operator |·| means in this case the absolute value. The multiplication with $h(\pi_{1},\pi_{2})$ is necessitated by cases in which inconsistent possibility distributions would otherwise be deemed redundant. Consider (11) without consistency ($1-|g(\mathrm{fu}(\pi_{1},\pi_{2}),\pi_{2})|$) and take, for example, two triangular possibility distributions $\pi_{1},\pi_{2}$ with $\mathrm{spec}(\pi_{1})=\mathrm{spec}(\pi_{2})$ and $0<h(\pi_{1},\pi_{2})<1$. Let the distributions $\pi_{1},\pi_{2}$ be positioned on the frame of discernment so that $\mathrm{spec}(\mathrm{fu}(\pi_{1},\pi_{2}))=\mathrm{spec}(\pi_{1})=\mathrm{spec}(\pi_{2})$. In this example, no information is gained by fusing $\pi_{1}$ and $\pi_{2}$ and so $1-|g(\mathrm{fu}(\pi_{1},\pi_{2}),\pi_{2})|=1$. Information is definitely changed. This needs to be reflected in a type I redundancy metric. As a result of this, (11) is upper bounded by $h(\pi_{1},\pi_{2})$.

**Figure 4:**
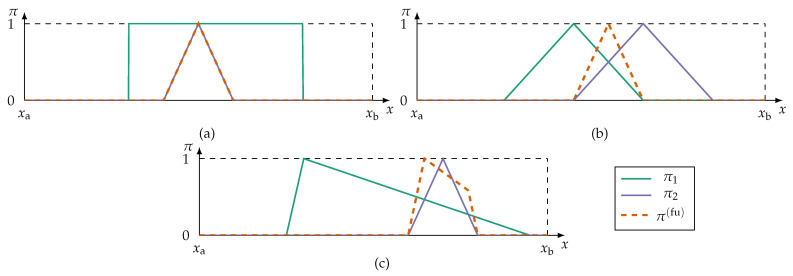
Possibility distributions and their fusion results as examples for the proposed type I redundancy metric. In (**a**), $r^{\left(I\right)}(\pi_{1},\pi_{2})=1$ and $0<r^{\left(I\right)}(\pi_{2},\pi_{1})<1$. Subfigure (**b**) shows a case in which both possibility distributions are not redundant, i.e., $0<r^{\left(I\right)}(\pi_{1},\pi_{2})<1$ and $0<r^{\left(I\right)}(\pi_{2},\pi_{1})<1$. Although the fusion result is less specific (more uncertain) in (**c**) due to renormalisation, both $\pi_{1}$ and $\pi_{2}$ are not redundant (similar to (**b**)).

Figure 4 shows examples of possibility distributions and their type I redundancy levels.

The degree of redundancy determined by (11) is dependent on how much the fusion changes the possibility distribution. Therefore, it is obvious that the choice of the fusion operator affects the redundancy measure. For the following propositions and proofs, it is assumed that fusion is carried out by applying the conjunctive fusion rule (4) if $h(\pi_{1},\pi_{2})>0$ and by applying the disjunctive fusion rule (5) if $h(\pi_{1},\pi_{2})=0$. The t-norm used for fusion is the minimum operator. Furthermore, possibility distributions are assumed to be normal.

**Proposition** **1.** *The metric $r^{\left(I\right)}$ (11) satisfies the boundaries property of Definition 3, i.e., it is bounded by [0,1].*

**Proof.**  The redundancy $r^{\left(I\right)}$ is based upon the information gain *g* (8) and the consistency of possibility distributions *h* (3). Both *g* and *h* are defined to be in [0,1]. It follows that $r^{\left(I\right)}\in \left[0,1\right]$. □

**Proposition** **2.** *The metric $r^{\left(I\right)}$ (11) satisfies the inclusion (upper bound) property of Definition 3, i.e., $r^{\left(I\right)}(\pi_{1},\pi_{2})=1\;if\;and\;only\;if\;\forall x\in X:\pi_{2}\left(x\right)\leq \pi_{1}\left(x\right)$.*

**Proof.**  For $r^{\left(I\right)}(\pi_{1},\pi_{2})=1$, $g(\pi_{1},\pi_{2})=0$ and $h(\pi_{1},\pi_{2})=1$. As a result that $\pi_{1}$ and $\pi_{2}$ are assumed to be normal and $\pi_{1}\left(x\right)\geq \pi_{2}\left(x\right)\;\forall x\in X$, $h(\pi_{1},\pi_{2})=1$. If $h(\pi_{1},\pi_{2})=1$, then $g(\pi_{1},\pi_{2})=0$ is only possible if either $\mathrm{fu}(\pi_{1},\pi_{2})=\pi_{1}$ or $\mathrm{fu}(\pi_{1},\pi_{2})=\pi_{2}$. Since $h(\pi_{1},\pi_{2})=1$, $\mathrm{fu}(\pi_{1},\pi_{2})=\frac{\min_{x\in X}(\pi_{1}(x),\pi_{2}(x))}{h(\pi_{1},\pi_{2})}=\min_{x\in X}(\pi_{1}(x),\pi_{2}(x))$. If $\pi_{1}\left(x\right)\geq \pi_{2}\left(x\right)\;\forall x\in X$, then $\min_{x\in X}(\pi_{1}(x),\pi_{2}(x))=\pi_{2}$. The information gain is then $g(\mathrm{fu}(\pi_{1},\pi_{2}),\pi_{2})=g(\pi_{2},\pi_{2})=0$, which implies that $r^{\left(I\right)}(\pi_{1},\pi_{2})=1$ iff $\forall x\in X:\pi_{2}\left(x\right)\leq \pi_{1}\left(x\right)$. □

An example of a fully redundant possibility distribution is shown in Figure 4a. If at least $\pi_{2}$ is subnormal or if other t-norms than the minimum operator are used, then $\mathrm{fu}(\pi_{1},\pi_{2})\neq \pi_{2}$. In this case the inclusion property is not strictly satisfied because $r^{\left(I\right)}(\pi_{1},\pi_{2})\gg 0$ instead of $r^{\left(I\right)}(\pi_{1},\pi_{2})=1$. In practical implementations it is still reasonable to apply other t-norms than the minimum operator, because the type I redundancy is still close to one.

**Proposition** **3.** *The metric $r^{\left(I\right)}$ (11) satisfies the lower bound property of Definition 3, i.e., $r^{\left(I\right)}(\pi_{1},\pi_{2})=0$ if $h(\pi_{1},\pi_{2})=0$. Additionally, $r^{\left(I\right)}(\pi_{1},\pi_{2})=0$ if $\pi_{2}$ models total ignorance and $\pi_{1}$ complete knowledge.*

**Proof.**  For $r^{\left(I\right)}(\pi_{1},\pi_{2})=0$ to be true, $g(\mathrm{fu}(\pi_{1},\pi_{2}),\pi_{2})=1$ or $h(\pi_{1},\pi_{2})=0$. Therefore, it is straightforward that $r^{\left(I\right)}(\pi_{1},\pi_{2})=0$ if $h(\pi_{1},\pi_{2})=0$. For $g(\mathrm{fu}(\pi_{1},\pi_{2}),\pi_{2})=1$, it needs to be true that $u\left(\pi_{2}\right)-u\left(\mathrm{fu}(\pi_{1},\pi_{2})\right)=1$ and $\mathrm{spec}\left(\mathrm{fu}(\pi_{1},\pi_{2})\right)-\mathrm{spec}\left(\pi_{2}\right)=1$. This is only true if (i) $\mathrm{spec}(\mathrm{fu}(\pi_{1},\pi_{2}))=1$ and (ii) $\mathrm{spec}(\pi_{2})=0$ because spec∈[0,1]. The latter requirement can be proven with (9). For $\mathrm{spec}(\pi_{2})=0$, $\int_{0}^{\alpha_{\max}}\left(\max_{x\in A_{\alpha }}x-\min_{x\in A_{\alpha }}x\right)\;d\alpha =\alpha_{\max}\cdot \left(x_{b}-x_{a}\right)$. This is only true if $A_{\alpha_{\max}}=X$ which is total ignorance. The first requirement can only be true if $\mathrm{fu}(\pi_{1},\pi_{2})$ represents complete knowledge per definition. The fusion of $\pi_{1}$ and $\pi_{2}$ can only result in complete knowledge if either $\pi_{1}$ or $\pi_{2}$ model complete knowledge. As a result that $\pi_{2}$ cannot represent both total ignorance and complete knowledge, $\mathrm{spec}(\pi_{1})=1$. □

**Proposition** **4.** *The metric $r^{\left(I\right)}$ (11) satisfies the identity property of Definition 3, i.e., $r^{\left(I\right)}(\pi_{1},\pi_{2})=1$ if $\pi_{1}=\pi_{2}$.*

**Proof.**  If $\pi_{1}=\pi_{2}$, then $g(\pi_{1},\pi_{2})=0$ and $h(\pi_{1},\pi_{2})=1$. It follows that $r^{\left(I\right)}(\pi_{1},\pi_{2})=1$. □

As defined in Definition 3, a redundancy metric should also yield meaningful results when more than two information items are involved, for instance if an information item is compared to a set of known information items. In such cases the fusion result is input into (11) instead of the single information items. Consider two sets of (unordered) information items $I_{1}=\left\{I_{1,1},I_{1,2},\ldots ,I_{1,n}\right\}$ and $I_{2}=\left\{I_{2,1},I_{2,2},\ldots ,I_{2,m}\right\}$ represented by possibility distributions. A set of information items I is different from an information source insofar that it is unordered. The items in the set I could be, for example, coming from several sources at the same instance. Given the sets $I_{1}$ and $I_{2}$, the redundancy is determined by

$$r^{\left(I\right)}\left(I_{1},I_{2}\right)=r^{\left(I\right)}(\mathrm{fu}(I_{1}),\mathrm{fu}(I_{2})).$$

For the design of information fusion systems, it is of interest which sensors or information sources form clusters with high internal redundancy. Identifying such clusters may be highly computational complex in large-scale multi-sensor systems. Given a large number of information sources, the following propositions may be helpful in reducing computational efforts. The proofs for these propositions are given in Appendix A.1. It is assumed that (i) fusion is carried out conjunctively (4) if $h(\pi_{1},\pi_{2})>0$ and disjunctively (5) if $h(\pi_{1},\pi_{2})=0$, (ii) that minimum and maximum operator fill the roles of t-norm and s-norm, and (iii) that all possibility distributions are normal.

**Proposition** **5.** *If $r^{\left(I\right)}(\pi_{1},\pi_{2})=1$ and $r^{\left(I\right)}(\pi_{2},\pi_{3})=1$, then $r^{\left(I\right)}(\pi_{1},\pi_{3})=1$.*

**Proposition** **6.** *Let $\pi_{1}$ and $\pi_{2}$ to be two possibility distributions which are fully consistent ($h(\pi_{1},\pi_{2})=1$). If $r^{\left(I\right)}(\pi_{3},\pi_{1})=1$ and $r^{\left(I\right)}(\pi_{3},\pi_{2})=1$, then $r^{\left(I\right)}(\pi_{3},\left\{\pi_{1},\pi_{2}\right\})=1$.*

**Corollary** **1.** *$r^{\left(I\right)}(\pi_{3},\left\{\pi_{1},\pi_{2}\right\})=1$ does not imply $r^{\left(I\right)}(\pi_{3},\pi_{1})=1$ or $r^{\left(I\right)}(\pi_{3},\pi_{2})=1$.*

**Proposition** **7.** *If $r^{\left(I\right)}(\pi_{1},\pi_{3})=1$ and $r^{\left(I\right)}(\pi_{2},\pi_{3})=1$, then $r^{\left(I\right)}\left(\left\{\pi_{1},\pi_{2}\right\},\pi_{3}\right)=1$.*

#### 4.1.2. Redundancy Type II

Redundancy Type I has been derived from the notion of fuzzy subsets and the change of specificity if new information items are considered. Redundancy Type II is more strict in the sense that information items are only considered to be redundant if they are similar, i.e., they convey the same information content. They are replaceable with each other without losing information in the process. In this respect, a set of information items is strictly similar if, in case of relying only on any single item, no information is lost at all and highly similar if only a small amount of information is lost.

Consequently, a type II redundancy measure which is set within possibility theory should be based on possibilistic similarity measures. Properties of such similarity measures have been given in [61,72,73], which define similarity to be a measure between only two possibility distributions. A definition adapted to sets of possibility distributions is proposed as follows:

**Definition** **5** (**Possibilistic Similarity Measure**)**.** *Let $p=\{\pi_{1},\pi_{2},\ldots ,\pi_{n}\}$ be an unordered set of possibility distributions defined on the same frame of discernment X. Then a possibilistic similarity measure is a function $\mathrm{sim}:\pi {\left(x\right)}^{n}\to \left[0,1\right]$ satisfying the following properties:*

- **Boundaries:** It is reasonable to assume that possibility distributions can be minimally and maximally similar. The measure sim(·) is therefore bounded. It is normalized if sim(p)∈[0,1].
- **Identity relation (upper bound):** A set of possibility distributions is maximally similar if they are identical, i.e., sim(π,π,…,π)=1 for any π. The reverse is not necessarily to be true. A set of possibility distributions with sim(p)=1 does not imply that all π∈p are identical.
- ***Non-agreement (lower bound):** The non-agreement property defines that any set of possibility distributions which cannot agree on a common alternative x to be possible are maximal dissimilar, i.e.,*
  sim(p)=0 if h(p)=0.
- ***Least agreement:** A set of possibility distributions p is at most as similar as the least similar pair $(\pi ,\pi^{\prime })\in p$:*
  $$\mathrm{sim}(p)\leq \min_{(\pi ,\pi^{\prime })\in p}(\mathrm{sim}(\pi ,\pi^{\prime })).$$
- **Symmetry:** A similarity measure is a symmetric function in all its arguments, that is, $\mathrm{sim}(\pi_{1},\pi_{2},\ldots ,\pi_{n})=\mathrm{sim}(\pi_{p(1)},\pi_{p(2)},\ldots ,\pi_{p(n)})$ for any permutation p on $N_{>0}$.
- **Inclusion:** For any $\pi_{1},\pi_{2},\pi_{3}$, if $\forall x\in X:\pi_{1}(x)\leq \pi_{2}(x)\leq \pi_{3}(x)$, then $\mathrm{sim}(\pi_{1},\pi_{3})\leq \mathrm{sim}(\pi_{1},\pi_{2})$ and $\mathrm{sim}(\pi_{1},\pi_{3})\leq \mathrm{sim}(\pi_{2},\pi_{3})$.

As a result of the intuitive closeness of Redundancy Type II to similarity measures, it is proposed that

(12) $$r^{\left(\mathrm{II}\right)}(p)=\mathrm{sim}(p).$$

All properties of type II redundancy metrics (Definition 4) are shared by similarity measures (Definition 5). Consequently, if a function is proven to be a similarity measure, then it is in the following not separately proven that it can function as a redundancy metric.

Similarity measures specifically designed towards possibility distributions have rarely been discussed until recently [61,72,73]. Before that, similarity of possibility distributions has been predominately determined either based on fuzzy set similarity measures or elementwise distance measurements. A short overview of the most important measures are given in the following. Advantages and disadvantages of measures regarding their application in multi-sensor systems are discussed.

One of the most simple possibilistic similarity measure satisfying the properties of Definition 5 is the consistency of possibility distributions:

(13) $$\mathrm{sim}(p)=h(p)=\max_{x\in X}(\min_{\pi \in p}(\pi (x))).$$

Proofs that consistency satisfies the properties of Definition 5 are given in [61] for two possibility distributions (|p|=2). As a result that the consistency measure is a concatenation of the minimum and maximum operator, it is indiscriminate to the number of information items. Therefore it satisfies the properties for |p|>2 also. Its simple nature is also its disadvantage. The consistency of possibility distributions is largely independent of shape or specificity producing unintuitive results if, e.g., given π and $\pi^{\prime }$ with $\mathrm{spec}(\pi )\gg \mathrm{spec}(\pi^{\prime })$. The most extreme example involves two normal possibility distributions representing total ignorance and complete knowledge, respectively, so that spec(π)=0 and $\mathrm{spec}(\pi^{\prime })=1$. Consistency produces in this case $\mathrm{sim}(\pi ,\pi^{\prime })=1$. On the other hand, consistency is advantageous because of its scalability and robustness. Its computational complexity scales linearly with the number of information items. Consistency is more robust against possibility distributions coming from not fully reliable sources than more sophisticated measures which rely on shape or specificity. Slightly erroneous possibility distributions may not result in a strong deviation of sim because consistency remains high as longs as there is some agreement in p. Of course, if a source is strongly unreliable and, thus, an information item is strongly deviating (e.g., it claims π(v)=0 for the unknown true value *v*) then consistency is also affected by this erroneous item.

Similarity is a more strict property than inclusion (as used for the Redundancy Type I). In terms of fuzzy set theory, two fuzzy sets A,B are similar if A⊆B and B⊆A. Two possibility distributions are, thus, completely similar ($\mathrm{sim}(\pi ,\pi^{\prime })=1$) if $\forall x\in X:\pi (x)\geq \pi^{\prime }(x)$ and $\pi^{\prime }(x)\geq \pi (x)$. Consequently, a similarity measure for the use as Redundancy Type II metric can be derived from (11). Considering the least-agreement requirement of Definition 5, taking the minimum $r^{\left(I\right)}$ of all pairwise combinations $(\pi ,\pi^{\prime })\in p$ creates a similarity measure:

(14) $${\mathrm{sim}}_{r}(p)=\min_{(\pi ,\pi^{\prime })\in p}(r^{\left(I\right)}(\pi ,\pi^{\prime })).$$

It is straightforward to proof that (14) satisfies all properties of Definition 5 (see Appendix A.2). However, (14) is computationally unfavourable since it (i) considers all pairwise combinations in p and (ii) it needs to compute the area beneath any π∈p and beneath any pairwise fusion results (see (8), (10), and (11)).

A widely practised approach is to adopt fuzzy set similarity measures—as they are—for possibility distributions. This seems reasonable because fuzzy sets and possibility distributions are defined mathematically very similarly (cf. Section 3.1). Most of the existing fuzzy similarity measures determine the overlap of fuzzy sets in different ways. For example, it has been proposed in several works (e.g., in [74]) to use the Jaccard index as a similarity measure (for an overview of fuzzy (dis-)similarity measures cf. [61,74]). Let *A* and *B* be two fuzzy sets, $\mu_{A}$ and $\mu_{B}$ their fuzzy membership functions, and ${\mathrm{sim}}_{\mu }$ be a fuzzy similarity measure, then the Jaccard index determines the similarity by ${\mathrm{sim}}_{\mu }(A,B)=\frac{A\cup B}{A\cap B}=\frac{\int_{x_{a}}^{x_{b}}\min(\mu_{A}(x),\mu_{B}(x))\;dx}{\int_{x_{a}}^{x_{b}}\max(\mu_{A}(x),\mu_{B}(x))\;dx}$. The direct possibilistic counterpart is then

(15) $$\mathrm{sim}(\pi_{1},\pi_{2})=\frac{\int_{x_{a}}^{x_{b}}\min(\pi_{1}(x),\pi_{2}(x))\;dx}{\int_{x_{a}}^{x_{b}}\max(\pi_{1}(x),\pi_{2}(x))\;dx}.$$

The Jaccard index is easily extended to more than two information items because it relies exclusively on intersection and union of fuzzy sets or minimum and maximum operators for possibility distributions. Equation (15) becomes then

(16) $$\mathrm{sim}(p)=\frac{\int_{x_{a}}^{x_{b}}\min_{\pi \in p}(\pi (x))\;dx}{\int_{x_{a}}^{x_{b}}\max_{\pi \in p}(\pi (x))\;dx}.$$

When using similarity measures based on fuzzy set theory it has to be kept in mind that fuzzy membership functions and possibility distributions do not convey the same meaning (as argued in Section 3.1). A membership function describes a fuzzy set completely. It is a mapping of elements to a degree of membership, i.e., it is known that v=x and *v* belongs to a fuzzy set with a degree of $\mu_{A}(x)$. In case of a possibility distribution, it is unknown whether v=x; it is only known that v=x is possible to π(x). Therefore, two non-overlapping fuzzy sets are two completely distinct entities. This motivates the non-agreement property (with regard to fuzzy sets: ${\mathrm{sim}}_{\mu }(A,B)$ if A∩B=0). There is a recent discussion ongoing whether the non-agreement property should be a requirement for possibilistic similarity measures [61]. The argument is that if there are two inconsistent possibility distributions $\pi_{1},\pi_{2}$ which are less distant apart in the frame of discernment than $\pi_{3},\pi_{4}$, then $\mathrm{sim}(\pi_{1},\pi_{2})>\mathrm{sim}(\pi_{3},\pi_{4})$. For that to be true, $\mathrm{sim}(\pi_{1},\pi_{2})$ would need to be greater than null, which does not conform with the non-agreement property.

A possibilistic similarity measure which does not adhere to the non-agreement property is based on information closeness [71] which is derived from the information gain (8):

(17) $$\begin{array}{c} G(\pi_{1},\pi_{2})=g(\pi_{1},\max_{x\in X}(\pi_{1}(x),\pi_{2}(x)))+g(\pi_{2},\max_{x\in X}(\pi_{1}(x),\pi_{2}(x))),\\ \mathrm{sim}(\pi_{1},\pi_{2})=1-\frac{G(\pi_{1},\pi_{2})}{2}. \end{array}$$

As a result that it is possible that $g(\pi_{1},\max_{x\in X}(\pi_{1}(x),\pi_{2}(x)))<1$ if $h(\pi_{1},\pi_{2})=0$, (17) does not satisfy the non-agreement property. Extending (17) to an indefinite number of possibility distribution (|p|>2) results in:

$$\begin{array}{c} G(p)=\sum_{\pi \in p}g(\pi ,\max_{x\in X,\pi^{\prime }\in p}(\pi^{\prime }(x))),\\ \mathrm{sim}(p)=1-\frac{G(p)}{\left|p\right|}. \end{array}$$

The non-agreement property is in accordance with the idea behind Redundancy Type II measures that each source or item in a redundant group carries the same information. Therefore, it is argued to implement redundancy metrics based on similarity measures which fulfil the non-agreement property.

#### 4.1.3. Reliability and Redundancy Metrics

**Figure 5:**
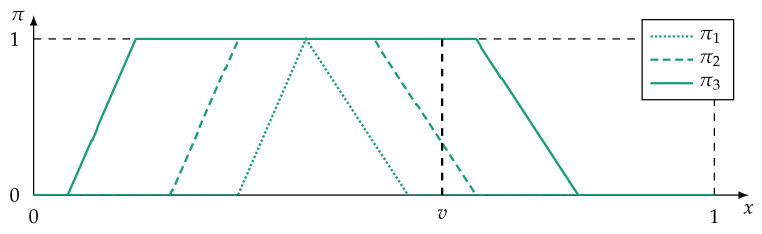
An incorrect ($\pi_{1}$), a partially erroneous ($\pi_{2}$), and a correct possibility distribution ($\pi_{3}$). The degree of error is dependent on the level of possibility πv, *v* being the unknown ground truth. Note that it is difficult to determine the error of a possibility distribution since *v* is unknown and it is precisely the task of π to give an imprecise estimation of *v*.

As pointed out in Section 1, unreliable information stems from defective information sources which experienced shifts, drifts, or produce outliers. Possibility distributions of unreliable sources tend to or actually give false estimations of the unknown value *v* (the ground truth). In the following a possibility distribution π is said to be strongly erroneous or incorrect if *v* lies outside of the crisp set *A* for which π gives support (v∉A, π(v)=0) and partially erroneous if v∈A but π(v)<1. Note that an unreliable source may provide incorrect possibility distributions but it does not necessarily need to do so, i.e., the source can still provide correct distributions. Figure 5 illustrates possibility distributions of different reliabilities. Identifying unreliable possibility distributions is a hard task because a possibility distribution is in itself an imprecise estimation of an unknown. Reliability assessments can be derived from knowledge about past behaviour (π may be unreliable or incorrect if a source as been proven to be unreliable in previous measurements) or by comparing π inside a group of sources known to be redundant. The reliability of a possibility distribution is inversely related with the quality of its information content (its specificity). The less specific a distribution is, the less likely it is to be erroneous. In the extreme case of total ignorance, a possibility distribution is completely free of error (and therefore reliable) but is of not much use since it is maximally non-specific.

Unreliable sources providing faulty possibility distributions may affect the proposed metrics for quantifying redundancy negatively. Shifted possibility distributions reduce the redundancy degree for both $r^{\left(I\right)}$ (11) and $r^{\left(\mathrm{II}\right)}$ (12), although it is argued that $r^{\left(\mathrm{II}\right)}$ is more easily and severely affected due to its stricter definition regarding similarity. Determining $r^{\left(I\right)}$ with (11) or $r^{\left(\mathrm{II}\right)}$ with (13) or (14), redundancy is lower if possibility distributions are inconsistent due to unreliable sources. Using (15) the overlap between distributions may be lower. More robust but not immune against occurring inconsistencies is (17).

As a result that a single faulty information item—even in large groups of items—can cause a drop in the determined redundancy, a preemptive method to increase robustness is desirable, especially for large multi-sensor systems. Let rel∈[0,1] be a reliability measure which states that an information source *S* is completely unreliable if rel(S)=0 and completely reliable if rel(S)=1. If rel(S) is known, then an approach to make use of this knowledge is to modify the information items, i.e., the possibility distribution, provided by *S* before they are processed further. The idea is to make a possibility distribution coming from an unreliable source less specific by widening or stretching it dependent on rel. Let $\pi^{\prime }$ be a modified possibility distribution based on π, then a widening modification function needs to satisfy the following properties:

- **Information preservation:** If rel=1, then the available information must not be changed but be preserved, i.e., $\pi^{\prime }=\pi$.
- **Specificity interaction:** If rel=0, then the information needs to be modified to model total ignorance, i.e., $\forall x\in X:\pi^{\prime }(x)=1$. Information must not get more specific by the modification: $\mathrm{spec}(\pi^{\prime })\geq \mathrm{spec}(\pi )$ for any rel∈[0,1].

A modification function has been proposed in [75] so that

(18) $$\pi^{\prime }(x)=\mathrm{rel}\cdot \pi (x)+1-\mathrm{rel},$$

and another in [13] so that

(19) $$\pi^{\prime }(x)=\max_{x\in X}(\pi (x),1-\mathrm{rel}).$$

**Figure 6:**
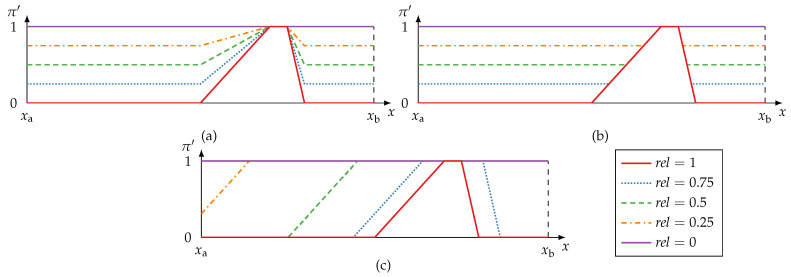
Modifying possibility distributions depending on the reliability of their information source *S*. Subfigure (**a**) shows the approach of Yager and Kelman (18), (**b**) shows the method of Dubois et al. (19), and (**c**) shows the proposed method (20). Only the method in (**c**) has a widening effect, both methods in (**a**,**b**) raise the level of possibility along the complete frame of discernment. All methods result in total ignorance for rel(S)=0 and $\pi^{\prime }=\pi$ for rel(S)=1. For these plots, parameter β=2.

Both modification functions raise the overall possibility level for all elements in the frame of discernment (see Figure 6 for an example). In this way, they stress the unpredictability of unreliable sources. Anything is possible in proportion to the unreliability of a source (1−rel(S)). This kind of approach towards modification functions is counterintuitive—especially in case of technical sensor systems—and leaves room for improvement. Consider, for instance, a sensor affected by drift due to ageing effects or due to environmental changes. In such a case, it is plausible that sensor readings are, e.g., slightly systematically off the true value or are affected by noise with an increasing amplitude. It is therefore more plausible that the unknown truth *v* is close to π(x) than that *v* is distant from π(x) (in an extreme case on the opposite side of the frame of discernment). For this reason, a modification function is proposed, which captures the essence of widening or stretching more closely, as follows:

(20) $$\begin{array}{c} \pi^{\prime }(x)=\max_{x^{\prime }\in C}(\pi (x^{\prime })),\\ C=\left[x-{\left(1-\mathrm{rel}\right)}^{\beta }\cdot \left(x_{b}-x_{a}\right),x+{\left(1-\mathrm{rel}\right)}^{\beta }\cdot \left(x_{b}-x_{a}\right)\right], \end{array}$$

with $x_{a}\in R$ being the minimum and $x_{b}\in R$ being the maximum border of *X*. Depending on rel and a control parameter $\beta \in R_{\geq 1}$, the modified possibility value $\pi^{\prime }(x)$ is the maximum possibility in the vicinity of *x*. This creates a widening effect. The parameter β provides an additional manual option to control the extent to which rel alters π(x). The larger β is chosen to be, the less effect rel has on π(x). For $\lim_{\beta \to \infty }$rel does not widen π(x).

The default value is β=1 in which case the unreliability has maximum effect in (20). It is straightforward to prove that (20) satisfies the requirements of information preservation and specificity interaction assuming π to be normal (see Appendix A.3). The proposed method of (20) is compared to the methods of [13,75] in Figure 6.

### 4.2. Redundant Information Sources

Up to this point, redundancy metrics for information items have been defined and discussed. A possibilistic redundancy metric for information sources is derived from $r^{\left(I\right)}$ and $r^{\left(\mathrm{II}\right)}$ in the following. It is defined as follows:

**Definition** **6** (**Possibilistic Redundancy Metric**)**.** *Let S be a possibilistic information source, i.e., the information items $I_{j}$ provided by S are possibility distributions: $I_{j}=\pi_{j}$ with $j\in N_{>0}$. Let S be the set of all available sources and P(S) be all possible combinations of sources, then a possibilistic redundancy metric ρ is a function which maps P(S) to the unit interval: ρ:P(S)→[0,1].*

*The metric ρ is derived from $r^{\left(\mathrm{II}\right)}$ (12). The following relations between ρ and $r^{\left(\mathrm{II}\right)}$ hold:*

- *If information sources are redundant, then they provide redundant information items. Consequently, ρ(S) increases as the redundancy of information items belonging to the sources in S increase.*
- *The reverse is not necessarily true. Redundant information items do no necessitate that their information sources are also redundant. Due to cases of incomplete information, redundant information items may support spurious redundancy (similar to spurious correlation which is depicted in Figure 1).*

*In this context and to qualify as an intuitively meaningful metric, the following requirements have to be met:*

- ***Boundaries:** A redundancy metric should be able to model complete redundancy and complete non-redundancy. It follows that ρ is minimally and maximally bounded. It is proposed that ρ∈[0,1].*
- ***Symmetry:** The metric ρ is a symmetric function in all its arguments, i.e.,*
  $$\rho \left(S_{1},S_{2},\ldots ,S_{j}\right)=\rho \left(S_{p(1)},S_{p(2)},\ldots ,S_{p(j)}\right)$$
*for any permutation p on $N_{>0}$.*

The possibilistic redundancy metric is proposed to be a function of two pieces of evidence. The evidence against redundancy $e_{c}:P(S)\to \left[0,1\right]$ captures the idea that redundant information items do not necessarily mean redundant information sources. The evidence $e_{c}$ is derived from $r^{\left(\mathrm{II}\right)}$: As long as information items are redundant, $e_{c}(S)=0$. It is discussed more closely in Section 4.2.1. Evidence in favour of redundancy $e_{p}:P(S)\to \left[0,1\right]$ is supposed to tackle the challenge of incomplete information. It indicates to which degree information is available from the complete frame of discernment. The evidence $e_{p}$ is discussed more closely in Section 4.2.2. A set of information sources is only redundant if $e_{p}(S)>0$ and $e_{c}(S)<1$. The smaller value of $e_{p}$ and the complement $1-e_{c}$ dominates the redundancy metric. The geometric mean is proposed as an averaging function for $e_{p}$ and $e_{c}$ as follows:

(21) $$\rho (S)=\rho (e_{c}(S),e_{p}(S))=\sqrt{e_{p}(S)\cdot \left(1-e_{c}(S)\right)}.$$

By splitting ρ into two separate evidences, it is aimed to achieve a cautious, more transparent metric.

#### 4.2.1. Evidence Against Redundancy

The measure $e_{c}$ indicates whether there is evidence that information sources are not redundant. In this sense, sources are assumed to be redundant as long as they are not proven to be otherwise (the complement of $e_{c}$ contributes to (21)). With regard to the redundancy metric $r^{\left(\mathrm{II}\right)}$ for information items, sources are evidenced to be non-redundant if they provide non-redundant items. Information sources are defined to be a set of ordered information items (see Definition 2). In order to derive $e_{c}$ from $r^{\left(\mathrm{II}\right)}$, an averaging function over the ordered items of sources is required. In the following, the short notation $r=r^{\left(\mathrm{II}\right)}$ is used.

Let $S=\{S_{1},S_{2},\ldots ,S_{n}\}$, i.e., let S be a set of information sources. Let each $S_{i}$ with i∈{1,⋯,n} provide an ordered set of possibility distributions ($S_{i}=\left\{\pi_{i,1},\pi_{i,2},\cdots ,\pi_{i,m}\right\}$), all of the same cardinality *m*. Let $p_{j}$ be the set of possibility distributions provided at the same instance *j*, i.e., $p_{j}=\left\{\pi_{1,j},\pi_{2,j},\ldots ,\pi_{n,j}\right\}$ (each source provides a single item to $p_{j}$), then

(22) $$e_{c}(S)=1-\underset{j=\{1,\ldots ,m\}}{\mathrm{avg}}(r(p_{j})).$$

The function avg(·) in the context of averaging redundancy values is a mapping ${\left[0,1\right]}^{m}\to \left[0,1\right]$. Averaging functions are required to be symmetric, idempotent, continuous, and increasingly monotone. Definitions of these properties can be found in [27,68]. Additionally, averaging functions satisfy the following inequality:

$$\min_{j=\{1,\ldots ,m\}}(r(p_{j}))\leq \underset{j=\{1,\ldots ,m\}}{\mathrm{avg}}(r(p_{j}))\leq \max_{j=\{1,\ldots ,m\}}(r(p_{j})).$$

Averaging functions which are closer to the minimum operator are said to be more *and-like*, whereas functions closer to the maximum operator are said to be more *or-like*.

The choice of the averaging function has a significant impact on $e_{c}$ and ultimately on the possibilistic redundancy metric (21). The mindset behind possibility theory—any world is possible unless shown otherwise (see [50] or Section 3.3)—is most closely realized if avg(·) satisfies the property of

- **Absorbing element:**$\mathrm{avg}(r(p_{1}),\ldots ,r(p_{m}),0)=0$ for any p, that is, if information sources in S produce non-redundant items, then this is evidence that S are not redundant as well.

Averaging functions which satisfy this property are the minimum operator $\min_{j\in \{1,\ldots ,m\}}(r(p_{j}))$ and the geometric mean $\sqrt[{m}]{\prod_{j=1}^{m}r(p_{j})}$. If information sources are known to or tend to producing outliers, then the absorbing element property results very easily in ${\mathrm{avg}}_{j=\{1,\ldots ,m\}}(r(p_{j}))=0$ and $e_{c}(S)=1$. Thus, minimum and geometric mean are only reasonable to apply, if sources are known to be reliable or if the effects of unreliable sources have been reduced by widening the possibility distributions (20). This requires the degree of reliability to be known or at least to be estimated. Comparing minimum and geometric mean, the geometric mean is less prone to unreliable sources. Although both satisfy the absorbing element property, the geometric mean is less strict in penalizing the occurrence of partially redundant items.

The arithmetic mean $\frac{1}{m}\sum_{j=1}^{m}r(p_{j})$ does not satisfy the absorbing element property and is not dominated by the minimum of its argument (and neither by the maximum). It is therefore more robust against unreliable sources, but it thwarts the basic idea that a possibilistic redundancy metric is supposed to handle incomplete or biased information. Consider a condition monitoring example, in which data represent predominately the system’s normal condition. In this example, this normal condition dominates the arithmetic mean and evidence against redundancy is neglected. This argument weighs even more heavily for all averaging functions which are more or-like than the arithmetic, such as the quadratic mean.

A controllable compromise between minimum dominated functions and arithmetic mean is to apply the class of ordered weighted averaging operators (OWA) [56]. OWA operators allow to control the degree of orness of an averaging function. Let $w=\{w_{1},\cdots ,w_{m}\}$ be an ordered set of weights with $w_{j}\in \left[0,1\right]$ and $\sum_{j=1}^{m}w_{j}=1$, then an OWA operator is

(23) $${\mathrm{avg}}_{\mathrm{OWA}}(p_{j},w)=\sum_{j=1}^{m}w_{j}\cdot r_{\left(j\right)}(p_{j}).$$

For OWA operators, the arguments (here: redundancies *r*) have to be ordered regarding their values in decreasing order. Therefore, $r_{\left(\cdot \right)}$ denotes a permutation such that $r_{\left(1\right)}\geq r_{\left(2\right)}\geq \ldots \geq r_{\left(m\right)}$. The orness orn:w→[0,1] of an OWA operator is defined by

$$\mathrm{orn}(w)=\frac{1}{m-1}\cdot \sum_{j=1}^{m}\left(m-j\right)\cdot w_{j}.$$

An OWA operator becomes the minimum operator if its orn(w)=0, i.e., w={0,0,…,1} and it becomes the arithmetic mean if $\mathrm{orn}(w)=\frac{1}{2}$, i.e., $w=\{\frac{1}{m},\frac{1}{m},\ldots ,\frac{1}{m}\}$. For a meaningful $e_{c}$, it is argued that $0\leq \mathrm{orn}(w)<\frac{1}{2}$. A method to compute weights w from orn(w) is given in [27,76]. The choice of orn(w) needs to be made carefully depending on knowledge about the application at hand (regarding incompleteness, bias of information) and the characteristics of applied information sources (regarding reliability).

#### 4.2.2. Evidence Pro Redundancy

**Figure 7:**
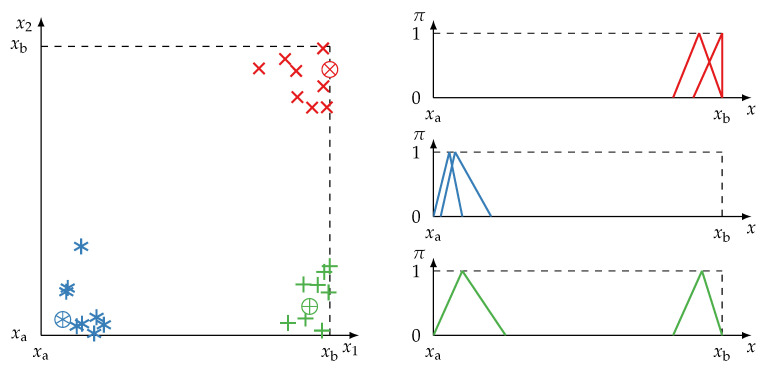
Information items in the form of triangular possibility distributions provided by two information sources. Available (e.g., measured) information is scattered throughout the frame of discernment $X=[x_{a},x_{b}]$. The left side shows a two-dimensional scatter plot in which each marker represents the maximum of each possibility distribution. The right side depicts the possibility distributions of three exemplary selected datapoints (marked by an encompassing circle). Each cluster considered in isolation represents a case of incomplete information because only parts of the frame of discernment are covered. For example, cluster 1 (marked by ×) suggests redundancy (as long as information items are similar). This may not hold when new information from both sources become available. Clusters 1 (×) and 2 (✶) together suggest redundancy more strongly. Any data containing cluster 3 (+) evidences no redundancy. Relying esclusively on $e_{c}$ (22) may result in detecting redundancy prematurely. A second evidence measure is needed to put $e_{c}$ into context. This second measure—denoted as evidence pro redundancy $e_{p}$—is presented in the following.

The second consideration to be made in constructing a redundancy metric is incomplete information on information item level (biased or skewed data). A technical system monitored by several information sources may not operate in all its possible states evenly. A cyber–physical production system may even exclusively run in its (intended) normal operation state; data gathered from faulty states may be rare or non-existent. For example, let the frame of discernment be all possible measurements from a sensor in all possible states of the monitored system. Assume that a system can be in an abnormal and a normal state. If a sensor observes the system only in its normal state, then the provided information items (i.e., possibility distributions) cover only a part of the frame of discernment, that is, the part which represents the normal state. Applying in this example the evidence contra redundancy measure $e_{c}$ (22), correlation coefficients, or mutual information may lead to premature redundancy detection. Premature redundancy is the case if information is redundant given the observed part of the frame of discernment, but not regarding the complete frame of discernment. Figure 7 illustrates cases of incomplete information motivating a second evidence measure which puts $e_{c}$ into context.

This second evidence $e_{p}$ quantifies how completely the available information covers the frame of discernment *X*. This coverage of *X* is in the following denoted as

**Definition** **7** (**Range**)**.** *Given a frame of discernment $X=[x_{a},x_{b}]$, the range of a set of possibility distributions p quantifies how far p stretches over X. Let P(p) bet the power set of al possible p, then the range is described by a function rge:P(p)→[0,1] with the following properties:*

- **Upper bound:** If rge(p)=1, then $\exists \pi \in p:\pi (x_{a})=1$ and $\exists \pi \in p:\pi (x_{b})=1$.
- **Lower bound:**rge(p)=0 if $\forall \pi ,\pi^{\prime }\in p:\pi =\pi^{\prime }$, that is, all possibility distributions π∈p are identical.

The range of available information is based on the position of a possibility distribution on the frame of discernment. The position is determined via the center of gravity [77]

(24) $$\mathrm{pos}(\pi )=\left\{\begin{array}{cc} x & \mathrm{if}\;\pi (x)=1\;\mathrm{and}\;\forall x^{\prime }\in \left\{X\backslash x\right\}:\pi (x^{\prime })=0,\\ \frac{\int_{x_{a}}^{x_{b}}x\cdot \pi (x)\;dx}{\int_{x_{a}}^{x_{b}}\pi (x)\;dx} & \mathrm{otherwise}. \end{array}\right.$$

Interesting properties of (24) for the determination of the range are:

- if ∀x∈X:π(x)=1 (π models total ignorance), then $\mathrm{pos}(\pi )=\frac{1}{2}\cdot \left(x_{b}-x_{a}\right)$,
- $\mathrm{pos}(\pi )=x_{a}$ if and only if $\pi (x_{a})=1$ and $\forall x\in \{X\backslash x_{a}\}:\pi (x)=0$ (π models complete knowledge at $x_{a}$), and
- $\mathrm{pos}(\pi )=x_{b}$ if and only if $\pi (x_{b})=1$ and $\forall x\in \{X\backslash x_{b}\}:\pi (x)=0$ (π models complete knowledge at $x_{b}$).

The position of a set of possibility distributions p is obtained by fusing the distribution prior to taking the center of gravity. Thus,

pos(p)=pos(fu(p)).

Let $(\pi ,\pi^{\prime })\in p$ denote all pairwise combinations of possibility distribution in p, then

(25) $$\mathrm{rge}(p)=\max_{(\pi ,\pi^{\prime })\in p}(\left|\mathrm{pos}(\pi )-\mathrm{pos}(\pi^{\prime })\right|)=\max_{\pi \in p}(\mathrm{pos}(\pi ))-\min_{\pi \in p}(\mathrm{pos}(\pi )).$$

Proofs that (25) satisfy the properties of Definition 7 are given in Appendix A.4.

Given a set of information sources $S=\{S_{1},S_{2},\ldots ,S_{n}\}$ in which $S_{i}=\left\{\pi_{i,1},\pi_{i,2},\cdots ,\pi_{i,m}\right\}$ and given that $p_{j}=\left\{\pi_{1,j},\pi_{2,j},\ldots ,\pi_{n,j}\right\}$, then

(26) $$\mathrm{rge}(S)=\max_{j,j^{\prime }\in \left\{1,\ldots ,m\right\}}(|\mathrm{pos}(p_{j})-\mathrm{pos}\left(p_{j^{\prime }}\right)|)=\max_{j\in \{1,\ldots ,m\}}(\mathrm{pos}(p_{j}))-\min_{j\in \{1,\ldots ,m\}}(\mathrm{pos}(p_{j})).$$

The range rge(S) quantifies the maximum distance of possibility distributions provided by S. At least one pair $p_{j},p_{j^{\prime }}$ of information item sets need to range over the frame of discernment *X* in order to provide evidence for a redundant behaviour, i.e., $e_{p}(S)>0$ if $\exists j\in \left\{1,\ldots ,m\right\}:\mathrm{rge}(p_{j})>0$. The range is normalized and then directly employed as evidence pro redundancy:

(27) $$e_{p}(S)=\frac{\mathrm{rge}(S)-x_{a}}{x_{b}-x_{a}}.$$

As a result that the range is derived from the position measure, rge(S)=1 iff $\exists j\in \left\{1,\ldots ,m\right\}:\mathrm{pos}(p_{j})=1$ and $\exists j\in \left\{1,\ldots ,m\right\}:\mathrm{pos}(p_{j})=0$ which is in accordance with the upper bound property of Definition 7. Therefore, only cases of complete knowledge result in rge=1. The lower bound rge(S)=0 iff $j,j^{\prime }\in \left\{1,\ldots ,m\right\}:\mathrm{fu}(p_{j})=\mathrm{fu}\left(p_{j^{\prime }}\right)$. The behaviour of rge in case of total ignorance is also noteworthy. Assume two information sources $S_{1},S_{2}$ providing total ignorance at all instances. Therefore, all possibility distributions of $S_{1},S_{2}$ are completely similar and the evidence against redundancy $e_{c}(S_{1},S_{2})=0$. Although they are similar, both sources have deemed all alternatives in *X* completely possible—they did not commit to any x∈X. There is no evidence or information that, if the sources will commit to alternatives in the future, both will commit to the same alternative and behave redundantly. Both sources have provided total ignorance up until the most recent instance, therefore $\mathrm{rge}(S_{1},S_{2})=0$, and, thus, no information is evident pro redundant behaviour. In this example, $e_{p}$ balances $e_{c}$ and helps to make a more well-grounded decision.

The evidences $e_{p}$ and $e_{c}$ form together a redundancy metric ρ which is cautious in cases of incomplete information. The proposed redundancy metric ρ is applicable to groups of information sources of any size. It quantifies how strongly a group of sources is redundant. It does not give information about whether there are redundant sources in this group, but rather if all sources in the complete group are redundant. In the following, it is proven that ρ (21) is a redundancy metric in accordance wit Definition 6.

**Proposition** **8.** *The proposed possibilistic redundancy metric ρ (21) satisfies the boundaries property of Definition 6, i.e., ρ(S)∈[0,1].*

**Proof.**  The metric ρ takes the geometric mean of $e_{p}(S)$ and $1-e_{c}(S)$. The geometric mean does not alter boundaries, so ρ∈[0,1] if $e_{p}\in \left[0,1\right]$ and $e_{c}\in \left[0,1\right]$.

- **$e_{p}$:** The evidence $e_{p}$ (27) is build upon the function rge (26) which in turn is build upon the function pos (24). The position $\mathrm{pos}\in [x_{a},x_{b}]$ because it is based on the center of gravity. The range rge takes the difference of maximum and minimum positions and is, therefore, also in $\left[x_{a},x_{b}\right]$. The evidence $e_{p}$ normalizes rge to the interval [0,1] in (27).
- **$e_{c}$:** The evidence $e_{c}$ (22) averages the redundancies of information items obtained by $r^{\left(\mathrm{II}\right)}$ which is by definition in [0,1] (see Definition 4).

□

**Proposition** **9.** *The proposed possibilistic redundancy metric ρ (21) satisfies the boundaries property of Definition 6, i.e., $\rho \left(S_{1},S_{2},\ldots ,S_{j}\right)=\rho \left(S_{p(1)},S_{p(2)},\ldots ,S_{p(j)}\right)$ for any permutation p on $N_{>0}$.*

**Proof.**  The metric ρ is symmetric if $e_{p}$ and $e_{c}$ are symmetric.

- $e_{p}$: The function range rge (26) takes the difference of the maximum and minimum of all $p_{j}$. the order of the information sources has no effect on rge. Equation (27) only normalizes rge. Thus, $e_{p}$ is symmetric.
- **$e_{c}$:** The type II redundancy metric $r^{\left(\mathrm{II}\right)}$ is symmetric per definition (Definition 4). The evidence $e_{c}$ (22) averages $r^{\left(\mathrm{II}\right)}$ over all provided information items and is consequently also symmetric.

□

## 5. Evaluation

The proposed possibilistic redundancy metric is evaluated qualitatively considering three datasets from technical application domains. Considered information sources are to a certain extent unreliable, provide noisy data, provide (un-)correlated data, and some perceive only a fraction of the frame of discernment. The redundancy metric ρ (21) is calculated for pairs of information sources (in the following also referred to as features). The metric is compared to to the Pearson’s correlation coefficient measure and an inconsistency-based approach—as identified in Section 2—with a strong focus on the correlation coefficient. The aim of this evaluation is to gain an understanding of the metric’s performance on practical data.

The evaluation is carried out on the Typical Sensor Defects (TSD) dataset [5], the Smartphone Dataset for Human Activity Recognition in Ambient Assisted Living (HAR) [78], and the Sensorless Drive Diagnosis (SDD) dataset [79]. The TSD dataset contains data obtained from a condition monitoring application of a storage container for hazardous and flammable substances. Applied sensors are, e.g., temperature sensors, smoke detectors, and gas detectors. The gathered data belongs exclusively to the normal condition of the observed system. Data is provided with an error of ±2% of the sensor’s measurement range creating a uniform probability density function. The TSD dataset is a set of datasets from which the dataset without sensor errors is used. Data in the HAR dataset tend to be affected by noise due to the low quality of applied sensors (smartphone sensors). The HAR dataset contains 6 classes, of which the activities *walking*, *walking upstairs* and *walking downstairs* are defined here as normal conditions. In the SDD dataset, a drive motor is examined for potential faults in the drive’s bearing. Sensors measure the voltage and current of the motor. The SDD dataset contains highly linearly correlated data. Both the HAR and SDD dataset provide data as precise singletons. Together, the TSD, HAR, and SDD datasets provide typical scenarios and challenges for data exploration. All three datasets are publicly available. The TSD dataset is uploaded and published by the authors of [5] (https://zenodo.org/record/56358 (accessed on 7 February 2021)). The SDD and HAR datasets are publicly available at the University of California Machine Learning Repository [80]. An overview of the selected datasets and their characteristics is given in Table 1.

### 5.1. Implementation

The datasets being considered in this evaluation do not provide possibility distributions. They contain several heterogeneous sensors as information sources. In general, information obtained from multi-sensor systems often need to be preprocessed due to any or all of the following reasons:

- Imprecision is modelled with probability distributions or not at all rather than with possibility distributions. Precise information items given as singletons are often only allegedly so—modelling the imprecision is often neglected.
- Information comes from unreliable sources.
- Information comes from heterogeneous sensors meaning that information is provided regarding different frame of discernments.

For each information item in a dataset the following preprocessing steps are therefore carried out:

1. If information are provided as singletons or probability distributions, they are transformed into possibility distributions.
2. The unreliability of information sources is taken into account by modifying (widening) the possibility distribution using (20) with parameters rel and β selected appropriately for each dataset.
3. All information are mapped to a common frame of discernment.

Step 1 of probability possibility transformation and step 3 regarding harmonizing the frame of discernments are detailed in the following sections. Modifying the possibility distributions is implemented with reliability parameters ∀S∈S:rel(S)=0.98 and β=1 for datasets SDD and TSD. For the HAR dataset ∀S∈S:rel(S)=0.85 and β=1 reflecting the poor quality of sensors in this dataset. Furthermore, the redundancy metric is implemented using the consistency measure (3) as similarity measure (see Definition 5) and using the geometric mean for the averaging of item-based redundancies (22), that is,

$$e_{c}(S)=1-\sqrt[{m}]{\prod_{j=1}^{m}r(p_{j})}.$$

#### 5.1.1. Probability Possibility Transform

If the imprecision of information is modelled with probability distributions, then a necessary preprocessing step is to transform the information into possibility distributions. A probability-possibility transformation is required to satisfy the following three conditions.

- **Normalization condition:** The resulting possibility distribution is required to be normal (∃x∈X:π(x)=1).
- **Consistency principle:** What is probable must preliminarily be possible, that is, the possibility of an event *A* is an upper bound for its probability (Pr(A)≤Π(A)).
- **Preference preservation:** Given a probability distribution *p*, $p\left(x\right)<p\left(x^{\prime }\right)\to \pi \left(x\right)\leq \pi \left(x^{\prime }\right)$.

A transformation is optimal if it loses as little information as possible in the transformation (following the maximum specificity principle). Dubois et al. [64] have proposed an optimal transform with regard to this principle. This optimal transform is for practical implementation purposes highly computationally complex and cumbersome to handle [66]. Therefore, the truncated triangular probability-possibility transform (TTPPT) is applied in this implementation which has been devised in [53,65,66]. The TTPPT is an approximation of an optimal transform which is less computationally complex. It can be applied to Gaussian, Laplace, triangular, and uniform probability density functions resulting in a truncated triangular possibility distribution. A truncated triangular possibility distribution is defined by three parameters $x_{n}\in X$, $x_{\epsilon }\in X$, and ϵ∈[0,1] as follows:

(28) $$\pi (x)=\left\{\begin{array}{cc} 0 & \mathrm{if}|x-x_{m}|>x_{n},\\ \epsilon & \mathrm{if}x_{n}\geq \left|x-x_{m}\right|>x_{\epsilon },\\ 1-\frac{1-\epsilon }{x_{\epsilon }}\cdot \left|x-x_{m}\right| & \mathrm{if}|x-x_{m}|\leq x_{\epsilon }. \end{array}\right.$$

Let p(x) be a probability density function (PDF), $x_{m}$ be the expected value of p(x), and σ its standard deviation, then $x_{n}$, $x_{\epsilon }$, and ϵ are determined depending on the type of PDF as listed in Table 2 (values obtained in [65]).

#### 5.1.2. Unifying Heterogeneous Information

Data in multi-sensor systems are often heterogeneous, i.e., data representing different physical quantities (e.g., voltage and electric current), or data in different dimensions (e.g., a scalar value and a vector). To be able to draw conclusions about the redundancy of heterogeneous sources, data are transformed into a unitless, uniform frame of discernment. A natural way to unify the frame of discernments is to make use of fuzzy sets. Given a binary or multi-class classification task, the fuzzy set representing each class can be exploited to transform the frame of discernments. In the following a binary classification task is assumed. The procedure is then to take a class, to model it with a fuzzy membership function μ, and, given x∈X, π(x), and μ(x), to compute the possibilities $\pi_{\mu }$ for each μ.

The membership function is implemented using a parametric, trainable unimodal potential function [81] defined by

(29) $$\begin{array}{c} \mu \left(x\right)=\left\{\begin{array}{c} 2^{-d(x,p_{l})}\mathrm{if}x\leq \bar{x},\\ 2^{-d(x,p_{r})}\mathrm{if}x>\bar{x}, \end{array}\right. \end{array}$$

$$\begin{array}{c} \mathrm{with}d\left(x,p_{l}\right)={\left(\frac{\left|x-\bar{x}\right|}{C_{l}}\right)}^{D_{l}}\mathrm{and}\\ d\left(x,p_{r}\right)={\left(\frac{\left|x-\bar{x}\right|}{C_{r}}\right)}^{D_{r}}. \end{array}$$

Unimodal potential functions were proposed by Aizerman et al. [82] as a tool for pattern recognition. It was not until later that they were applied in the fuzzy set community as membership functions [14,81]. Unimodal potential functions are used to model the distribution of compact objects of convex classes [14]. The function parameters allow to asymmetrically adjust the function to the distribution of a class which are either determined by training data or by expert’s knowledge. The advantages of unimodal potential functions are that their parameters are both simple to learn and intuitively to interpret.

In dataset TSD, the parameters for (29) are provided, which are determined by an expert. For datasets SDD and HAR, the parameters are obtained as follows. Let $x=\{x_{1},x_{2},\ldots ,x_{n}\}$ be the available training data, then parameter $\bar{x}$ is the arithmetic mean of x. Parameter $C_{l}=\bar{x}-\min_{i\in \{1,2,\ldots ,n\}}(x_{i})$ and $C_{r}=\max_{i\in \{1,2,\ldots ,n\}}\left(x_{i}\right)-\bar{x}$. Parameters $D_{l}$, $D_{r}\in N_{>1}$. In state-of-the-art applications they are often determined empirically such as in [5,83]. In [84], a method to learn parameters $D_{l}$ and $D_{r}$ is proposed based on density estimations of the training data. In the implementation of this work, $D_{l}=2$ and $D_{r}=2$ for all datasets TSD, HAR, and SDD. Due to its parametric and trainable character, the unimodal potential function and its variations have shown to be particularly effective in practice—from industrial [5,83] to medical applications [85].

As a final step, the possibility distributions π(x), x∈X, are transformed to $\pi_{\mu }(\mu (x))$, μ∈[0,1]. The transformation is carried out by applying the unimodal potential function (29) as follows:

(30) $$\pi_{\mu }(\mu )=\max_{x\in \{X\;|\;\mu (x)=\mu \}}(\pi (x)).$$

Note that, due to the bell shape of the potential function, μ is a non-injective but surjective mapping. In (29) the same membership is assigned to two different *x* (with the exception of $\mu (\bar{x})=1$ which is unique). This necessitates the maximum operator in (30).

**Figure 8:**
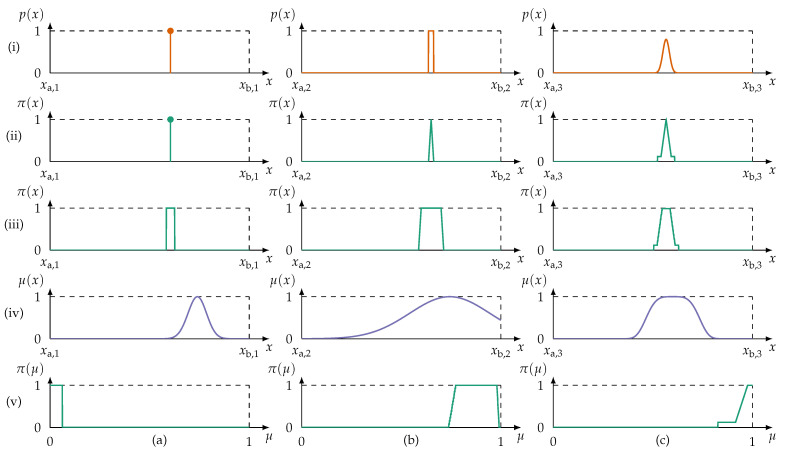
Preprocessing steps (i)–(v) carried out on three information items provided as probability distributions p(x)—as (**a**) singular value, (**b**) uniform probability density function, and (**c**) Gaussian probability density function. Each item gives information regarding an unknown measurand in its own frame of discernment ($X_{1}=\left[x_{a,1},x_{b,1}\right]$, $X_{2}=\left[x_{a,2},x_{b,2}\right]$, $X_{3}=\left[x_{a,3},x_{b,3}\right]$). As a result of this, preprocessing is necessary to be able to derive conclusions about potential redundancy. First, in step (ii) the probability distributions are transformed into possibility distributions via the truncated triangular probability-possibility transformation [53,65,66]. Step (iii) takes account of potential unreliability of information sources by widening π(x) using (20) (here with rel=0.95 and β=1). Steps (iv), (v) transform the frame of discernment into fuzzy memberships $X_{\mu }=\left[\mu_{a},\mu_{b}\right]=\left[0,1\right]$. Assuming a binary fuzzy classification task, one fuzzy class (e.g., the normal condition in condition monitoring) is represented by a unimodal potential function (UPF) (29) either learned from training data or provided by an expert (iv) (here: arbitrary selected UPFs are shown as an example). Whereas π(x) in (iii) represents the imprecision of a single information item, μ(x) represents the fuzzy set of the given class. In the final step (v), π(x) is transformed into π(μ) (30). Note that π(x) aligns with μ(x) in such a way that (**a**) π(μ) is close to 0 and (**b**), (**c**) π(μ) is close to 1.

The complete preprocessing sequence is exemplary illustrated in Figure 8 with information provided as a singleton, as a uniform PDF, and as a Gaussian PDF.

### 5.2. Results and Discussion

**Figure 9:**
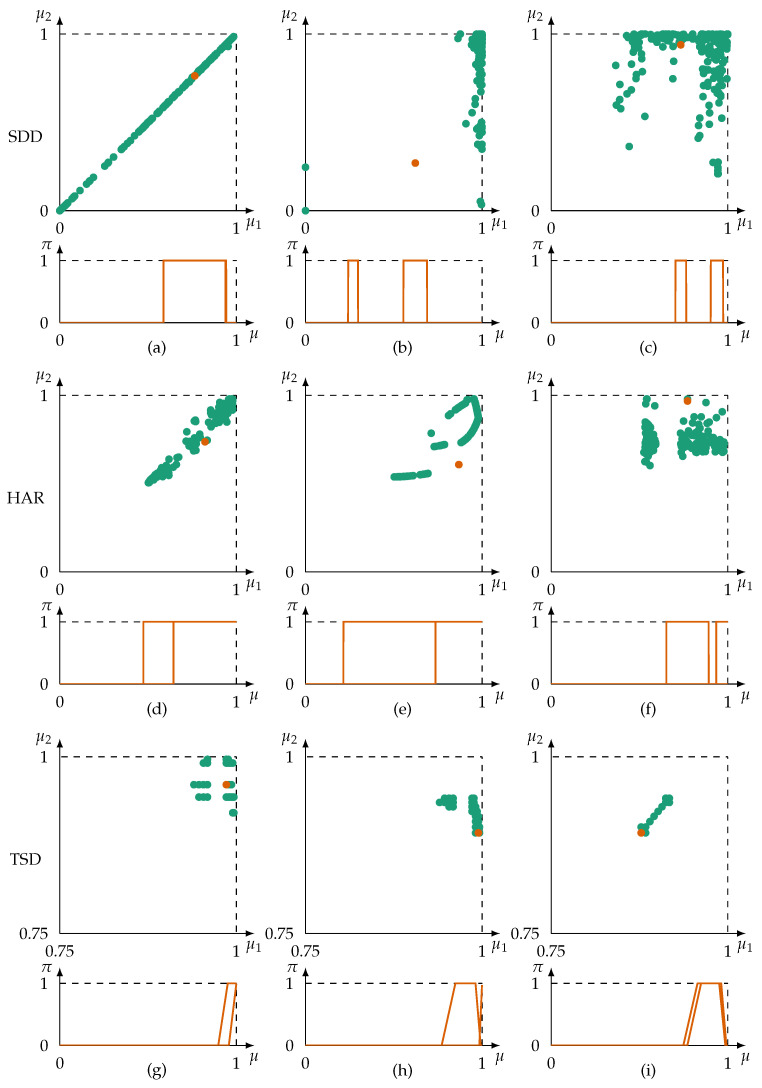
Information items of the selected information sources. Each row, consisting of a scatter and linear plot, belongs to sources from the datasets Sensorless Drive Diagnosis (SDD) (**a**–**c**), HAR (**d**–**f**), and Typical Sensor Defects (TSD) (**g**–**i**). Each point in the scatter plots represents the center of gravity (24) of an information item, i.e., of $\pi_{\mu }(\mu (x))$. To get an intuition about the imprecision in the information, the possibility distributions of a single pair of information items are plotted below each scatter plot. The selected cases show linear relations, non-linear relations, non-redundancy, and aleatoric noise. In some only part of the frame of discernment is perceived. Note that plots (**g**–**i**) are zoomed in for better visibility.

For the qualitative evaluation, information sources are selected from the datasets which exhibit different types of relations and provide different challenges for determining their redundancies. Sources are either linearly correlated, non-linearly correlated, non-redundant, affected by aleatoric noise, or are a combination thereof. In addition, the perceptive fields of sources are limited to varying proportions of the frame of discernment (information is biased or incomplete). Redundancy is computed only for pairs of sources. Selected pairs of information sources are: (a) 7 and 8 from SDD, (b) 2 and 46 from SDD, (c) 20 and 36 from SDD, (d) 86 and 99 from HAR, (e) 89 and 102 from HAR, (f) 12 and 50 from HAR, (g) 9 and 15 from TSD, (h) 9 and 18 from TSD, and (i) 14 and 18 from TSD. Information coming from the selected sources are illustrated for all cases (a)–(i) in Figure 9.

In Section 2 state-of-the-art measures for quantifying redundancy between information sources are identified. These are (i) the Pearson’s correlation coefficient $\rho_{p}$ (probabilistic), (ii) mutual information (probabilistic), (iii) inconsistency (possibilistic; used as a measure for non-redundancy) [5], and conflict (based on Dempster–Shafer theory; used as a measure for non-redundancy) [46,47]. Of these, the proposed redundancy metric is evaluated against $\rho_{p}$ and the approach based on inconsistency. Mutual information measures the degree of dependency between probability distributions based on their entropy. The probability distribution from which data is generated needs to be known in advance. Given the datasets, these distributions are precisely not known. Considering that data are real-valued in the selected datasets, probability distributions cannot be constructed ad hoc based on frequency of occurrences (which is more reasonable if data are categorical or integer-valued). This dilemma regarding MI shows its lack of practicability given applications with unknown probability distributions of data and is the reason why it is excluded here.

The following list provides details about the implementation of compared approaches for the sake of reproducibility:

- **Pearson’s correlation coefficient:** Correlation coefficients are computed on the expected value of the original data because sources from the TSD dataset provide information associated with an imprecision interval modeled by a uniform PDF. Let $x_{i,j}^{\left(e\right)}$ be the expected value of the imprecise data provided by source $S_{i}$ at instance *j* and let ${\bar{x}}_{i}^{\left(e\right)}$ be the arithmetic mean of the expected values of $S_{i}$. Then, the correlation coefficient is computed by
  $$\rho_{p}\left(S_{1},S_{2}\right)=\left|\frac{\sum_{j=1}^{m}\left(x_{1,j}^{\left(e\right)}-{\bar{x}}_{1}^{\left(e\right)}\right)\left(x_{2,j}^{\left(e\right)}-{\bar{x}}_{2}^{\left(e\right)}\right)}{\sqrt{\sum_{j=1}^{m}{\left(x_{1,j}^{\left(e\right)}-{\bar{x}}_{1}^{\left(e\right)}\right)}^{2}\sum_{i=1}^{m}{\left(x_{2,j}^{\left(e\right)}-{\bar{x}}_{2}^{\left(e\right)}\right)}^{2}}}\right|.$$
- **Inconsistency-based approach:** In [5] the inconsistency inc of a possibility distribution is determined within a set of possibility distributions. The inconsistency is the distance between the distribution’s position pos(π) and the position of the majority observation $\mathrm{pos}(\pi_{\mathrm{maj}})$ within the set: $\mathrm{inc}=\left|{\mathrm{pos}}_{\pi }-\mathrm{pos}(\pi_{\mathrm{maj}})\right|$. The position is determined by (24). Since we compare only pairs of information sources, no majority observation can be found and the distance between the positions of both information items is taken. The approach in [5] is designed for streaming data and the inconsistency of information items is averaged with a moving average filter. Instead of this kind of filter, inc is averaged so that:
  $$\mathrm{inc}(S_{1},S_{2})=\frac{1}{m}\cdot \sum_{j=1}^{m}\left|\mathrm{pos}(\pi_{1,j})-\mathrm{pos}(\pi_{2,j})\right|.$$
  Similar to our approach, a homogenous frame of discernment between information items is required. Therefore, the inconsistency is computed on the possibility distributions $\pi_{\mu }$ obtained by the preprocessing steps detailed previously. The measure inc determines the degree of non-redundancy between information sources.

Results including ρ, $e_{p}$, $e_{c}$, $\rho_{p}$, and inc are shown for each case in Table 3.

The possibilistic redundancy metric quantifies the redundancy of information in the presented cases differently. The metric itself conveys more sophisticated information about the relation between sources than the correlation coefficient. The metric ρ quantifies the linear case (a) of the SDD dataset as highly redundant. Information items are both assessed as similar ($e_{c}\ll 1$) and range over a significant part of the frame of discernment ($e_{p}\gg 0$). Case (d)—linear with noise—and case (e)—non-linear—of the HAR dataset show both highly similar items as well. As a result that information is limited to only a part of the frame of discernment, ρ=0.69. In cases (b), (c), and (f), there is high evidence that the sources are not redundant ($e_{c}=1$). The overall metric is dominated by the minimum of 1−$e_{c}$ and $e_{p}$, therefore ρ=0. All pairs of information sources coming from the TSD dataset (g)–(i) are highly similar. Therefore, there is little evidence that they are not redundant ($e_{c}\ll$ 1). In these cases, information sources perceive only a small part of the frame of discernment. Consequently, their range is close to 0. There is also close to no evidence that sources are redundant. A reasonable interpretation is that sources may be redundant but that more information is required to consolidate the claim of redundancy. Thus, the split of the possibilistic redundancy metric into $e_{p}$ and $e_{c}$ makes it possible to assess redundancy relations in more detail. Relying only on similarity measures would in these cases lead to premature identifications of redundancies. This would negatively impact applications in machine learning as well as information fusion.

In comparison to the Pearson’s correlation coefficient, the possibilistic metric is more cautious in suggesting redundancy. This is most evident in cases (d), (e), and (g)–(i) in which $\rho_{p}$ assigns higher values than ρ. This is because ρ does not solely rely on similarity measures but also on the range of information (on which $e_{p}$ is based). The correlation coefficient does not have this kind of point of reference. This is especially problematic in case (i) in which sources show correlated behaviour but, because the information covers only a small specific part of the frame of discernment, it cannot be said with certainty that they are truly redundant. In the other cases, the values of ρ and $\rho_{p}$ are more close. It stands out that ρ tends to assign zero redundancy more easily. The coefficient $\rho_{p}$ determines correlation statistically, whereas ρ takes non-agreeing information as evidence against redundancy. This leads ρ to reject redundancy faster.

The inconsistency-based approach requires some expert’s knowledge to interpret correctly. Higher values of inc suggest non-redundant behaviour of information sources. Regarding cases (c) and (f), in which information is deemed as non-redundant by both ρ and $\rho_{p}$, inc>0.1. Using this value as a threshold, it is noteworthy that the inconsistency-based approach does not detect non-redundancy in case (b). Similar to $\rho_{p}$ it does not take contextual information regarding the frame of discernment into account. In summary, inc is less intuitively to read and results in less correct estimations.

## 6. Conclusions

Redundancy takes a key role in the robustness of algorithms and models applied to intelligent technical multi-sensor systems. Redundant information sources serve as a back-up in case of malfunctioning sensors, but also allow to detect drifts more easily. Standard existing approaches that determine redundancy between information sources, for instance correlation coefficients or information-theoretical metrics, do not take into account epistemic uncertainties such as incomplete or imprecise information.

This article contributes a redundancy metric set in the framework of possibility theory. It explicitly determines redundancy between information sources which provide imprecise information, e.g., in the form of probability or possibility distributions. Redundancy of sources is determined based on two evidential measures. The first determines whether single imprecise information items are redundant in principle—either based on inclusion or similarity measures. If all items of sources are redundant, then the sources are deemed potentially redundant. The second evidence measure—based on the range of information—determines whether sufficient information is available to consolidate the first evidence. This results in a cautious redundancy metric which does not assign high redundancy values in case of incomplete information. In contrast, metrics based on correlation coefficients may detect redundancies prematurely.

A most important aspect of implementations for large-scale technical and cyber–physical systems are the scalability and computational complexity of methods. It is simply not feasible to assess every subset of the available information sources regarding their redundancy since the power set of sources grows exponentially with each added source. In order to ensure scalability, two steps have to be approached in future work. First, the possibilistic redundancy metric needs to be analysed and optimized regarding its computational complexity. Second, methods and strategies have to be designed which analyse the power set of sources in a clever way—either by relying on expert’s knowledge or by reducing the search space based on deductions from already analysed sources.

Additionally, in technical systems data are streamed, that is, only a limited amount of data are available in advance if at all. Algorithms need to be able to cope with streaming data, adapt to new information, and update previous knowledge incrementally. The possibilistic redundancy metric is engineered to be cautious on available information with the idea that it can be updated as soon as new information becomes available. The proposed metric still has to be analysed regarding its updatability, i.e., whether it needs to be computed from scratch for every new information or whether it can be updated.

## Figures and Tables

**Table 1:** Overview of the selected datasets.

Dataset	Information Sources (Columns)	Information Items (Rows)	Format	Imprecision	Noteworthy Characteristics
SDD	48	58509	real-valued, x∈R	precise, px=v=1, px≠v=0	highly linearly correlated
HAR	561	5744	real-valued, x∈R	precise, px=v=1, px≠v=0	noisy
TSD	22	72500	real-valued, x∈R binary-valued, x∈{0,1}	imprecise, uniform PDF	incomplete information

**Table 2:** Parameters $x_{n}$, $x_{\epsilon }$, and ϵ for the truncated triangular probability-possibility transform of different probability density functions (PDF).

PDF	$x_{n}$	$x_{\epsilon }$	ϵ
Gaussian	2.58·σ	1.54·σ	0.12
Laplace	3.20·σ	1.46·σ	0.13
Triangular	2.45·σ	1.63·σ	0.11
Uniform	1.73·σ	1.73·σ	0

**Table 3:** Results of the possibilistic redundancy metric ρ (21) along with the evidences $e_{p}$ (27) and $e_{c}$ (22). The metric is compared to (i) the Pearson’s correlation coefficient $\rho_{p}$ computed on the expected values of the original data and an inconsistency-based approach (measure inc). The cases (a)–(i) refer to the selected information sources as plotted in  Figure 9.

Case	Dataset	$S_{1}$	$S_{2}$	$e_{p}$	$e_{c}$	ρ	$\rho_{p}$	inc
(a)	SDD	7	8	0.92	0	0.96	1	0
(b)	SDD	2	46	0.85	1	0	0.09	0.06
(c)	SDD	20	36	0.76	1	0	0.07	0.19
(d)	HAR	89	102	0.47	0	0.69	0.98	0.02
(e)	HAR	86	99	0.47	0	0.69	0.94	0.09
(f)	HAR	12	50	0.47	1	0	0.27	0.16
(g)	TSD	9	15	0.04	0	0.20	0.90	0.16
(h)	TSD	9	18	0.04	0.10	0.19	0.89	0.02
(i)	TSD	14	18	0.05	0	0.23	0.99	0.02

## Data Availability

Not applicable.

## Abbreviations

The following abbreviations are used in this manuscript:

CPS	Cyber–Physical Systems
MI	Mutual Information
OWA	Ordered Weighted Averaging
PDF	Probability Density Function
PosT	Possibility Theory
ProbT	Probability Theory
TTPPT	Truncated Triangular Probability-Possibility Transform
UPF	Unimodal Potential Function

## Appendix A. Additional Proofs

### Appendix A.1. Proofs of Section 4.1.1

In all following proofs, the short notation $\pi \geq \pi^{\prime }$ is used instead of ∀x∈X:π(x)$\geq \pi^{\prime }\left(x\right)$.

In Proposition 5, it is stated that if $r^{\left(I\right)}(\pi_{1},\pi_{2})=1$ and $r^{\left(I\right)}(\pi_{2},\pi_{3})=1$, then $r^{\left(I\right)}(\pi_{1},\pi_{3})=1$.

**Proof** **of** **Proposition** **5.** $r^{\left(I\right)}(\pi ,\pi^{\prime })=1$ iff $\pi \geq \pi^{\prime }$ [57]. If $\pi_{1}\geq \pi_{2}\geq \pi_{3}$, then $\pi_{1}\geq \pi_{3}$ and $r^{\left(I\right)}(\pi_{1},\pi_{3})=1$. □

Assuming $\pi_{1}$ and $\pi_{2}$ to be two possibility distributions which are fully consistent ($h(\pi_{1},\pi_{2})=1$), it is to be proven that Proposition 6 holds, i.e., if $r^{\left(I\right)}(\pi_{3},\pi_{1})=1$ and $r^{\left(I\right)}(\pi_{3},\pi_{2})=1$, then $r^{\left(I\right)}(\pi_{3},\left\{\pi_{1},\pi_{2}\right\})=1$.

**Proof** **of** **Proposition** **6.** First, $r^{\left(I\right)}(\pi ,\pi^{\prime })=1$ iff $\pi \geq \pi^{\prime }$ [57]. If $h(\pi_{1},\pi_{2})=1$, then $\mathrm{fu}(\pi_{1},\pi_{2})=\frac{\min(\pi_{1},\pi_{2})}{h(\pi_{1},\pi_{2})}=\min(\pi_{1},\pi_{2})$. Since $\pi_{3}\geq \pi_{1}$ and $\pi_{3}\geq \pi_{2}$, $\pi_{3}\geq \min(\pi_{1},\pi_{2})$. It follows that $r^{\left(I\right)}(\pi_{3},\left\{\pi_{1},\pi_{2}\right\})=1$. □

Corollary 1 states that $r^{\left(I\right)}(\pi_{3},\left\{\pi_{1},\pi_{2}\right\})=1$ does not imply $r^{\left(I\right)}(\pi_{3},\pi_{1})=1$ or $r^{\left(I\right)}(\pi_{3},\pi_{2})=1$.

**Proof** **of** **Corollary** **1.** As a result that fusion is carried out by a t-norm (in case of $h(\pi_{1},\pi_{2})>0$), no information about the shape of neither $\pi_{1}$ or $\pi_{2}$ can be induced (other than they overlap because $h(\pi_{1},\pi_{2})>0$). Therefore, it cannot be implied that $r^{\left(I\right)}(\pi_{3},\pi_{1})=1$ or $r^{\left(I\right)}(\pi_{3},\pi_{2})=1$. □

**Proof** **of** **Proposition** **7.** It is to be proven that if $r^{\left(I\right)}(\pi_{1},\pi_{3})=1$ and $r^{\left(I\right)}(\pi_{2},\pi_{3})=1$, then $r^{\left(I\right)}(\left\{\pi_{1},\pi_{2}\right\},\pi_{3})=1$. In both cases of conjunctive and disjunctive fusion, it holds that if both $\pi_{1}\geq \pi_{3}$ and $\pi_{2}\geq \pi_{3}$, then $\mathrm{fu}(\pi_{1},\pi_{2})\geq \pi_{3}$. Therefore, $r^{\left(I\right)}(\left\{\pi_{1},\pi_{2}\right\},\pi_{3})=1$. □

### Appendix A.2. Proofs of Section 4.1.2

**Proposition** **A1.**
*The possibilistic similarity measure ${\mathrm{sim}}_{r}$ proposed in (14) satisfies the properties of Definition 5.*

**Proof** **of** **Boundaries** **Property.** A possibilistic similarity measure ${\mathrm{sim}}_{r}$ is defined to be in [0,1]. Since $r^{\left(I\right)}\in \left[0,1\right]$, $\min_{(\pi ,\pi^{\prime })\in p}(r^{\left(I\right)}(\pi ,\pi^{\prime }))\in \left[0,1\right]$ and ${\mathrm{sim}}_{r}\in \left[0,1\right]$. □

**Proof of Identity Relation** **(Upper** **Bound)** **Property.** Identity relation is satisfied if ${\mathrm{sim}}_{r}\pi ,\pi ,\ldots ,\pi =1$. Obviously, each pair in {π,π,…,π} has h(π,π)=1 and fu(π,π)=π. Thus, no information is gained in the fusion process: g(fu(π,π),π)=g(π,π)=0. Therefore, each pair has $r^{\left(I\right)}(\pi ,\pi )=1$. It follows that ${\mathrm{sim}}_{r}(\pi ,\pi ,\ldots ,\pi )=1$. □

**Proof of Non-Agreement** **(Lower** **Bound)** **Property.** Non-agreement is satisfied if ${\mathrm{sim}}_{r}(p)=0$ in case of h(p)=0. Since (11) includes a multiplication with *h*, all pairs $(\pi ,\pi^{\prime })\in p$ have $r^{\left(I\right)}(\pi ,\pi^{\prime })=0$ and, thus, ${\mathrm{sim}}_{r}(p)=0$. □

**Proof** **of** **Least** **Agreement** **Property.** Least agreement is satisfied if ${\mathrm{sim}}_{r}(p)\leq \min_{(\pi ,\pi^{\prime })\in p}$ $\left({\mathrm{sim}}_{r}(\pi ,\pi^{\prime })\right)$. Similarity between two possibility distributions is ${\mathrm{sim}}_{r}(\pi ,\pi^{\prime })=$ $\min(r^{\left(I\right)}(\pi ,\pi^{\prime }),r^{\left(I\right)}(\pi^{\prime },\pi ))$. Then, ${\mathrm{sim}}_{r}(p)=\min_{(\pi ,\pi^{\prime })\in p}(r^{\left(I\right)}(\pi ,\pi^{\prime }))=\min_{(\pi ,\pi^{\prime })\in p}$ $\left(\min(r^{\left(I\right)}(\pi ,\pi^{\prime }),r^{\left(I\right)}(\pi^{\prime },\pi ))\right)=\min_{(\pi ,\pi^{\prime })\in p}({\mathrm{sim}}_{r}(\pi ,\pi^{\prime }))$. □

**Proof** **of** **Symmetry** **Property.** The symmetry property is satisfied if ${\mathrm{sim}}_{r}(\pi_{1},\pi_{2},\ldots ,\pi_{n})=\mathrm{sim}(S_{p(1)},S_{p(2)},\ldots ,S_{p(n)})$ for any permutation *p* on $N_{>0}$. The similarity measure ${\mathrm{sim}}_{r}$ is symmetric because the minimum operator in (14) itself is symmetric and the minimum type I redundancy is taken from all possible pairwise combinations in $\left\{\pi_{1},\pi_{2},\ldots ,\pi_{n}\right\}$, e.g., ${\mathrm{sim}}_{r}\pi ,\pi^{\prime }=\min(r^{\left(I\right)}(\pi ,\pi^{\prime }),r^{\left(I\right)}(\pi^{\prime },\pi ))$. □

**Proof** **of** **Inclusion** **Property.** Inclusion is satisfied if (i) ${\mathrm{sim}}_{r}(\pi_{1},\pi_{3})\leq {\mathrm{sim}}_{r}(\pi_{1},\pi_{2})$ and (ii) ${\mathrm{sim}}_{r}(\pi_{1},\pi_{3})\leq {\mathrm{sim}}_{r}(\pi_{2},\pi_{3})$ in case of $\pi_{1}(x)\leq \pi_{2}(x)\leq \pi_{3}(x)\forall x\in X$. Let i,j∈{1,2,3}. Then $\forall i<j:r^{\left(I\right)}(\pi_{j},\pi_{i})=1$, $r^{\left(I\right)}(\pi_{i},\pi_{j})\leq 1$, and, therefore, ${\mathrm{sim}}_{r}(\pi_{i},\pi_{j})=\min(r^{\left(I\right)}(\pi_{j},\pi_{i}),r^{\left(I\right)}(\pi_{i},\pi_{j}))=r^{\left(I\right)}(\pi_{i},\pi_{j})$. Since $h(\pi_{i},\pi_{j})=1$, $\mathrm{fu}(\pi_{i},\pi_{j})=\pi_{i}$.

Part (i) ${\mathrm{sim}}_{r}(\pi_{1},\pi_{3})\leq {\mathrm{sim}}_{r}(\pi_{1},\pi_{2})$ if $r^{\left(I\right)}(\pi_{1},\pi_{3})\leq r^{\left(I\right)}(\pi_{1},\pi_{2})$. It follows that
  $$\begin{array}{cc} r^{\left(I\right)}(\pi_{1},\pi_{3}) & \leq r^{\left(I\right)}(\pi_{1},\pi_{2}),\\ \left(1-|g(\mathrm{fu}(\pi_{1},\pi_{3}),\pi_{3})|\right)\cdot h(\pi_{1},\pi_{3}) & \leq \left(1-|g(\mathrm{fu}(\pi_{1},\pi_{2}),\pi_{2})|\right)\cdot h(\pi_{1},\pi_{2}),\\ 1-|g(\mathrm{fu}(\pi_{1},\pi_{3}),\pi_{3})| & \leq 1-|g(\mathrm{fu}(\pi_{1},\pi_{2}),\pi_{2})|,\\ 1-|g(\pi_{1},\pi_{3})| & \leq 1-|g(\pi_{1},\pi_{2})|,\\ -|g(\pi_{1},\pi_{3})| & \leq -|g(\pi_{1},\pi_{2})|,\\ -|\underset{<0}{\underset{︸}{\mathrm{spec}(\pi_{1}(-\mathrm{spec}(\pi_{3})}}| & \leq -|\underset{<0}{\underset{︸}{\mathrm{spec}(\pi_{1})-\mathrm{spec}(\pi_{2})}}|,\\ \mathrm{spec}(\pi_{1})-\mathrm{spec}(\pi_{3}) & \leq \mathrm{spec}(\pi_{1})-\mathrm{spec}(\pi_{2}),\\ \mathrm{spec}(\pi_{3}) & \geq \mathrm{spec}(\pi_{2}). \end{array}$$
Part (ii) ${\mathrm{sim}}_{r}(\pi_{1},\pi_{3})\leq {\mathrm{sim}}_{r}(\pi_{2},\pi_{3})$ if $r^{\left(I\right)}(\pi_{1},\pi_{3})\leq r^{\left(I\right)}(\pi_{2},\pi_{3})$. The same steps as carried out in part (i) can be applied. This leads to
  $$\begin{array}{cc} -|\underset{<0}{\underset{︸}{\mathrm{spec}(\pi_{1})-\mathrm{spec}(\pi_{3})}}| & \leq -|\underset{<0}{\underset{︸}{\mathrm{spec}(\pi_{2})-\mathrm{spec}(\pi_{3})}}|,\\ \mathrm{spec}(\pi_{1})-\mathrm{spec}(\pi_{3}) & \leq \mathrm{spec}(\pi_{2})-\mathrm{spec}(\pi_{3}),\\ \mathrm{spec}(\pi_{1}) & \leq \mathrm{spec}(\pi_{2}). \end{array}$$

□

### Appendix A.3. Proofs of Section 4.1.3

**Proposition** **A2.**
*The proposed modification method in (20) satisfies the information preservation property, i.e., if rel=1, $\pi^{\prime }=\pi$.*

**Proof.** If rel=1, then ${\left(1-\mathrm{rel}\right)}^{\beta }\cdot \left(x_{b}-x_{a}\right)=0$. Consequently, C={x} and $\pi^{\prime }(x)=\max_{x^{\prime }\in \left\{x\right\}}(\pi (x^{\prime }))=\pi (x)$. □

**Proposition** **A3.**
*The proposed modification method in (20) satisfies the specificity interaction property, i.e., both (i) if rel=0, $\forall x\in X:\pi^{\prime }(x)=1$ and (ii) $\mathrm{spec}(\pi^{\prime })\geq \mathrm{spec}(\pi )$.*

**Proof.** Part (i) If rel=0, then ${\left(1-\mathrm{rel}\right)}^{\beta }\cdot \left(x_{b}-x_{a}\right)=\left(x_{b}-x_{a}\right)$ and *C* ranges ∀x over the complete frame of discernment, i.e., C=X. π is assumed to be normal, therefore $\pi^{\prime }(x)=\max_{x^{\prime }\in X}\pi (x^{\prime })=1\forall x\in X$. Part (ii) Equation (20) relies solely on the maximum operator. Therefore, $\pi^{\prime }(x)\geq \pi (x)$ and $\mathrm{spec}(\pi^{\prime })\geq \mathrm{spec}(\pi )$ (see (9) or (10)). □

### Appendix A.4. Proofs of Section 4.2.2

**Proposition** **A4.**
*Equation (25) satisfies the upper bound property of Definition 7.*

**Proof.** rge(p)=1 if $\max_{\pi \in p}(\mathrm{pos}(\pi ))-\min_{\pi \in p}(\mathrm{pos}(\pi ))=x_{b}-x_{a}$. As can be seen in (24), (i) $\max_{\pi \in p}(\mathrm{pos}(\pi ))=x_{b}$ if $\pi (x_{b})=1$ and $\forall x\in \{X\backslash x_{b}\}:\pi (x)=0$ and (ii) $\min_{\pi \in p}(\mathrm{pos}(\pi ))=x_{a}$ if $\pi (x_{a})=1$ and $\forall x\in \{X\backslash x_{a}\}:\pi (x)=0$. □

**Proposition** **A5.**
*Equation (25) satisfies the lower bound property of Definition 7.*

**Proof.** If $\forall \pi ,\pi^{\prime }\in p:\pi =\pi^{\prime }$, then $\mathrm{pos}(\pi )=\mathrm{pos}(\pi^{\prime })$ and max(pos(π))=min(pos(π)). It follows that rge(p)=0. □
